# Discovery of Novel Biomarker Candidates for Liver Fibrosis in Hepatitis C Patients: A Preliminary Study

**DOI:** 10.1371/journal.pone.0039603

**Published:** 2012-06-26

**Authors:** Bevin Gangadharan, Manisha Bapat, Jan Rossa, Robin Antrobus, David Chittenden, Bettina Kampa, Eleanor Barnes, Paul Klenerman, Raymond A. Dwek, Nicole Zitzmann

**Affiliations:** 1 Oxford Antiviral Drug Discovery Unit, Oxford Glycobiology Institute, Department of Biochemistry, University of Oxford, Oxford, United Kingdom; 2 Nuffield Department of Clinical Medicine, University of Oxford, Oxford, United Kingdom; 3 Oxford NIHR Biomedical Research Centre, The John Radcliffe Hospital, Headington, Oxford, United Kingdom; Duke University School of Medicine, United States of America

## Abstract

**Background:**

Liver biopsy is the reference standard for assessing liver fibrosis and no reliable non-invasive diagnostic approach is available to discriminate between the intermediate stages of fibrosis. Therefore suitable serological biomarkers of liver fibrosis are urgently needed. We used proteomics to identify novel fibrosis biomarkers in hepatitis C patients with different degrees of liver fibrosis.

**Methodology/Principal Findings:**

Proteins in plasma samples from healthy control individuals and patients with hepatitis C virus (HCV) induced cirrhosis were analysed using a proteomics technique: two dimensional gel electrophoresis (2-DE). This technique separated the proteins in plasma samples of control and cirrhotic patients and by visualizing the separated proteins we were able to identify proteins which were increasing or decreasing in hepatic cirrhosis. Identified markers were validated across all Ishak fibrosis stages and compared to the markers used in FibroTest, Enhanced Liver Fibrosis (ELF) test, Hepascore and FIBROSpect by Western blotting. Forty four candidate biomarkers for hepatic fibrosis were identified of which 20 were novel biomarkers of liver fibrosis. Western blot validation of all candidate markers using plasma samples from patients across all Ishak fibrosis scores showed that the markers which changed with increasing fibrosis most consistently included lipid transfer inhibitor protein, complement C3d, corticosteroid-binding globulin, apolipoprotein J and apolipoprotein L1. These five novel fibrosis markers which are secreted in blood showed a promising consistent change with increasing fibrosis stage when compared to the markers used for the FibroTest, ELF test, Hepascore and FIBROSpect. These markers will be further validated using a large clinical cohort.

**Conclusions/Significance:**

This study identifies 20 novel fibrosis biomarker candidates. The proteins identified may help to assess hepatic fibrosis and eliminate the need for invasive liver biopsies.

## Introduction

More than 170 million individuals, approximately 3% of the world’s population, are currently infected with the hepatitis C virus (HCV) [1]. Infection with HCV is one of the leading causes of liver fibrosis which, if left untreated, can develop into cirrhosis and hepatocellular carcinoma. The current reference standard for assessing hepatic fibrosis is liver biopsy followed by histological analysis [2]. This procedure is invasive, expensive and up to 40% of patients experience severe pain. Coupled with this, if hepatic fibrosis is not homogenous the rate of false negatives from liver biopsy can be as high as 20%, with sampling error observed when biopsies under 10 mm are analysed [2], [3].

Various non-invasive approaches have been proposed for assessing hepatic fibrosis including protein, glycoprotein and glycan biomarkers [2]. The FibroTest, a test based on five serum markers – apolipoprotein A1, haptoglobin, gamma glutamyltranspeptidase, alpha 2 macroglobulin and bilirubin, has been described to reduce the number of biopsies for managing HCV infection [4], but it eliminates the need for biopsy in only 26% of patients [5]. A more recent development, the Enhanced Liver Fibrosis (ELF) test, uses tissue inhibitor of metalloproteinase 1 (TIMP-1), hyaluronic acid and procollagen III amino terminal peptide (PIIIP) [6], [7]. PIIIP has low diagnostic value in assessing fibrosis [8] and both PIIIP and hyaluronic acid increase in patients with viral hepatitis after interferon alpha treatment [9]. Hepascore [10] (which uses bilirubin, gamma glutamyltranspeptidase, hyaluronic acid, alpha 2 macroglobulin) and FIBROSpect [11] (which uses hyaluronic acid, TIMP-1, and alpha 2 macroglobulin) are other fibrosis tests which use the same markers among FibroTest and ELF test. Although all these established tests are often able to discriminate between absence of fibrosis and advanced fibrosis/cirrhosis, serum markers have difficulties in classifying the intermediate stages between these two extremes often referred to as a ‘gray area’ [12]. In view of this, there remains a need for more reliable non-invasive markers to decrease the need for liver biopsy.

Previously we and others have used 2-DE over a wide pH 3–10 range to successfully identify several novel candidate biomarkers for liver fibrosis [13], [14]. In our previous proteomics study we show that all proteins which increased or decreased in expression in early or moderate fibrosis (Ishak stages 1–3) also change in cirrhosis, but not all proteins which increased or decreased in cirrhosis also change in the earlier fibrosis stages [13]. In this study we compare plasma samples from healthy control individuals with samples from patients with cirrhosis using a narrower pH range, and then test whether any of the proteins with significantly changed expression levels also show a consistent and quantifiable change in the intermediate ‘gray area’, which we analysed by Western blotting using plasma samples across all Ishak fibrosis scores. We have recently shown that 2-DE with a narrow pH 3–5.6 range is a novel approach which is beneficial for biomarker discovery [15], [16]. In the current study, we have increased our panel of candidate biomarkers for hepatic fibrosis by using this novel approach to compare plasma samples from healthy control individuals with samples from patients with cirrhosis. The pH 3–5.6 range was chosen since this lies outside the range of highly abundant albumin, transferrin and immunoglobulins. This enables more protein to be loaded than in our previous fibrosis marker study and enhances representation of low abundance proteins.

A selection of markers identified reliably changed in expression across all Ishak fibrosis scores when analysed using Western blotting and are novel candidates for non-invasive fibrosis markers. These markers appear to be very promising when compared to the markers in FibroTest, ELF test, Hepascore and FIBROSpect.

## Materials and Methods

### Patient Samples

Plasma samples were collected in P100 tubes (BD, Oxford, UK) from 50 subjects: 45 HCV-infected patients with varying degrees of hepatic fibrosis and 5 healthy control individuals. The patients were all recruited from outpatients attending for routine follow up visits at the John Radcliffe Hospital, Oxford, UK. The Ishak scores of all 45 patients were determined as previously described [17] and these scores along with other clinical details are displayed in Table S1. Collection of all plasma samples for this study was carried out by obtaining both verbal and written informed consent from each individual and this was approved by the Central Oxford Research Ethics Committee (No. 98.137). Unlike our previous study in which we used serum [13], for the current study we used plasma to discover novel fibrosis biomarkers, using recently developed P100 tubes. Unlike any other blood collection tube, this tube contains proprietary protein stabilizers that solubilize immediately as blood is collected and thus enhances preservation and recovery of proteins making them ideal for proteome analysis and biomarker discovery [18]. These samples were used for two studies: Firstly, plasma from healthy control individuals and patients with cirrhosis were compared by proteomics using 2-DE to identify candidate fibrosis biomarkers. Secondly, these candidate markers were validated by Western blotting using plasma samples from patients across all seven Ishak scores. All the blood samples used for both 2-DE and Western blotting were acquired within 12 months of liver biopsy except for three cirrhotic patients who had biopsies more than 12 months before sampling but were clinically cirrhotic and therefore biopsies within 12 months were not required (see Table S1).

### Proteomics using 2-DE with pH 3–5.6 Strips

Plasma samples from five different healthy control individuals and five different HCV-infected patients with hepatic cirrhosis (with an Ishak score [17] of 6) were initially selected for 2-DE analysis (Table 1). Samples were run by 2-DE using the approach we recently described [15], [16]. Two mg of the plasma samples were made up to 375 µl in isoelectric focusing (IEF) rehydration buffer (5 M urea, 2 M thiourea, 2 mM tributyl phosphine, 65 mM DTT, 4% (w/v) CHAPS, 150 mM non-detergent sulfobetaine 256 (NDSB-256) and 0.0012% (w/v) bromophenol blue) with 1.8% (v/v) pH 3–6 ampholytes (SERVALYT®, SERVA, Heidelberg, Germany). Samples were left overnight to rehydrate 18 cm pH 3–5.6 DryStrips (GE Healthcare, Bucks, UK). Isoelectric focusing was carried out for 75 kVh at 17°C. Strips were incubated in equilibration solution (4 M urea, 2 M thiourea, 50 mM Tris-HCl (pH 6.8), 30% (v/v) glycerol, 2% (w/v) SDS, 130 mM DTT, 0.002% (w/v) bromophenol blue) for 15 min. Proteins were separated by 9–16% (w/v) SDS-PAGE gradient gels using 20 mA per gel for 1 h, followed by 40 mA per gel for 4 h at 10°C. Following electrophoresis, gels were fixed in 40% (v/v) ethanol and 10% (v/v) acetic acid and stained with the fluorescent dye OGT 1238 [19]. Gels were scanned using an Apollo II linear fluorescence scanner (Oxford Glycosciences, Abingdon, UK) to obtain 16-bit images at 200 µm resolution.

**Table 1:** Details of the 20 plasma samples used for 2-DE and Western blotting.

Sample	Age	Sex	Ishak score	2-DE	Western blotting	MELD score for cirrhotic patients	Child-Pugh for cirrhotic patients
C1	28	F	0	Normal 1	Lane 1		
C2	41	F	0	Normal 2	Lane 2		
C3	47	M	0	Normal 3	Lane 3		
C4	28	M	0	Normal 4	Lane 4		
C5	56	M	0	Normal 5	na		
346	52	M	6	Cirrhosis 1	Lane 15	na	A
291	52	M	6	Cirrhosis 2	Lane 16	8	A
412	58	M	6	Cirrhosis 3	na	11	A
427	60	M	6	Cirrhosis 4	na	na	na
105	53	M	6	Cirrhosis 5	na	11	A
197	46	F	1	na	Lane 5		
146	49	M	1	na	Lane 6		
267	58	M	2	na	Lane 7		
446	42	F	2	na	Lane 8		
417	49	M	3	na	Lane 9		
440	46	F	3	na	Lane 10		
436	36	M	4	na	Lane 11		
447	71	M	4	na	Lane 12		
302	48	F	5	na	Lane 13		
191	57	M	5	na	Lane 14		

Sample name for 2-DE (in Figure S1) and lane number for Western blotting (in Figure 4) are shown.

na  =  not analysed.

Other clinical details for these samples and the other 30 plasma samples studied are in Table S1.

### Differential Image Analysis

Scanned gel images were processed with a custom version of the Melanie II software (Oxford Glycosciences, Abingdon, UK) [19]. For image analysis, five gels of plasma from different healthy control individuals were compared with five gels of plasma from different HCV-infected patients with hepatic cirrhosis. A synthetic image was created using accurate spot matching which showed all protein spots (features) in the gels for control and cirrhotic plasma. The optical density of each feature was determined by summing pixels within the feature boundary and the volume was determined by integrating this optical density over the area of the feature. All statistical calculations were based on the percentage volume of the features and changes in protein expression were determined as a ratio of the mean percentages of feature volumes. Since some low abundant features may be undetected by the algorithm of the software, features present in at least three of five individual gels belonging to either the control or cirrhotic group of gels were considered for statistical analysis and all gels were later visualised in a montage format to confirm if the features were present in all five gels. Features which changed in expression by at least 2-fold in percentage spot volume were considered as differentially expressed and were statistically validated using a rank-sum test on percentage spot volumes with *P* ≤ 0.05 (95% confidence) as previously described [19], [20]. All changes in expression were further validated by visualizing the differentially expressed features across all gels in a montage format and these features were excised from the gels for mass spectrometric analysis. All gels were calibrated using landmarks of known pH and molecular weight so that the pH and molecular weights of the differentially expressed features could be determined.

### In-gel Digestion and Peptide Extraction

Differentially expressed features assigned for mass spectrometric analysis were excised from gels using a software-driven robotic cutter (Oxford Glycosciences, Abingdon, UK). Recovered gel pieces were dried in a SpeedVac followed by in-gel trypsin digestion and peptide extraction using the automated DigestPro workstation (Intavis, Cologne, Germany) as we recently described [16]. Digested samples were lyophilised and dissolved in 0.1% (v/v) formic acid prior to mass spectrometric analysis.

### Mass Spectrometric Analysis

Tryptic peptides were analysed using a Q-TOF 1 mass spectrometer coupled to a CapLC (Waters, Hertfordshire, UK). Peptides were concentrated and desalted on a 300 µm I.D./5 mm C18 precolumn and resolved on a 75 µm I.D./25 cm C18 PepMap analytical column (LC packings, CA, USA) with a 45 min 5–95% (v/v) acetonitrile gradient containing 0.1% (v/v) formic acid at a flow rate of 200 nl/min. Spectra were acquired in positive mode. MS to MS/MS switching was controlled in an automatic data-dependent fashion with a 1 s survey scan followed by three 1 s MS/MS scans. Ions selected for MS/MS were excluded from further fragmentation for 2 min. Raw MS/MS spectra were smoothed and centred using ProteinLynx Global server 2.1.5, spectra were not deisotoped. Processed peak list (.pkl) files were searched against the SWISS-PROT database (release 56.9) using MASCOT Daemon 2.1.0 (Matrix Science, London, UK). Searches were restricted to human taxonomy (20402 sequences). Carbamidomethyl cysteine was defined as a fixed modification and oxidized methionine as a variable modification. Data were searched allowing 0.5 Da error to accommodate calibration drift and up to 2 missed tryptic cleavage sites. A minimum ion score cut-off of 28 was applied with confident protein assignment requiring a minimum of 2 unique peptides. The MSMS of all single peptide IDs were manually validated and required a minimum of 6 y-ions (or b-ions for C-terminal peptides.) MSMS spectra quality was judged by s/n, fragment ion isotopes and fragmentation markers such as proline residues.

### Biomarker Validation

Western blotting was used to validate the novel fibrosis markers identified and were compared to protein markers in FibroTest, ELF test, Hepascore and FIBROSpect also using Western blotting. Four different plasma samples from controls (Ishak score 0) and two different plasma samples from patients in each of the six Ishak stages of hepatic fibrosis (stages 1–6) were used for Western blotting (Table 1). These 16 samples were blotted for the markers in FibroTest, ELF test, Hepascore and FIBROSpect and our novel fibrosis markers. Immunoblotting was performed essentially as previously described. [20] Four plasma samples from controls (Ishak score 0) and two plasma samples from patients in each of the six Ishak stages of hepatic fibrosis (stages 1–6) were resolved on 17-well SDS-PAGE gels. Separated plasma proteins were electroblotted onto nitrocellulose membranes (Hybond ECL; GE Healthcare, Bucks, UK), blocked in 0.2% (w/v) casein for 2 h at room temperature, and probed successively with primary antibodies at 4°C and horseradish peroxidase (HRP) labeled secondary antibodies at room temperature. Bands were detected with Enhanced Chemiluminescence Plus reagent (ECL Plus) (GE Healthcare, Bucks, UK). The primary anti human antibodies and secondary HRP conjugated antibodies used for Western blotting are listed in Method S1. The non-protein marker hyaluronic acid was measured using a competitive ELISA and total bilirubin and gamma glutamyltranspeptidase were measured by the Biochemistry department in the John Radcliffe Hospital, Oxford (see Method S1).

### Ingenuity Pathways Analysis

The Ingenuity Pathways Analysis software (Ingenuity Systems, CA, USA) was used to investigate possible interactions between all proteins identified. Interactive pathways were generated to observe potential direct and indirect relations among the differentially expressed proteins.

## Results

### Identification of Candidate Biomarkers

**Figure 1:**
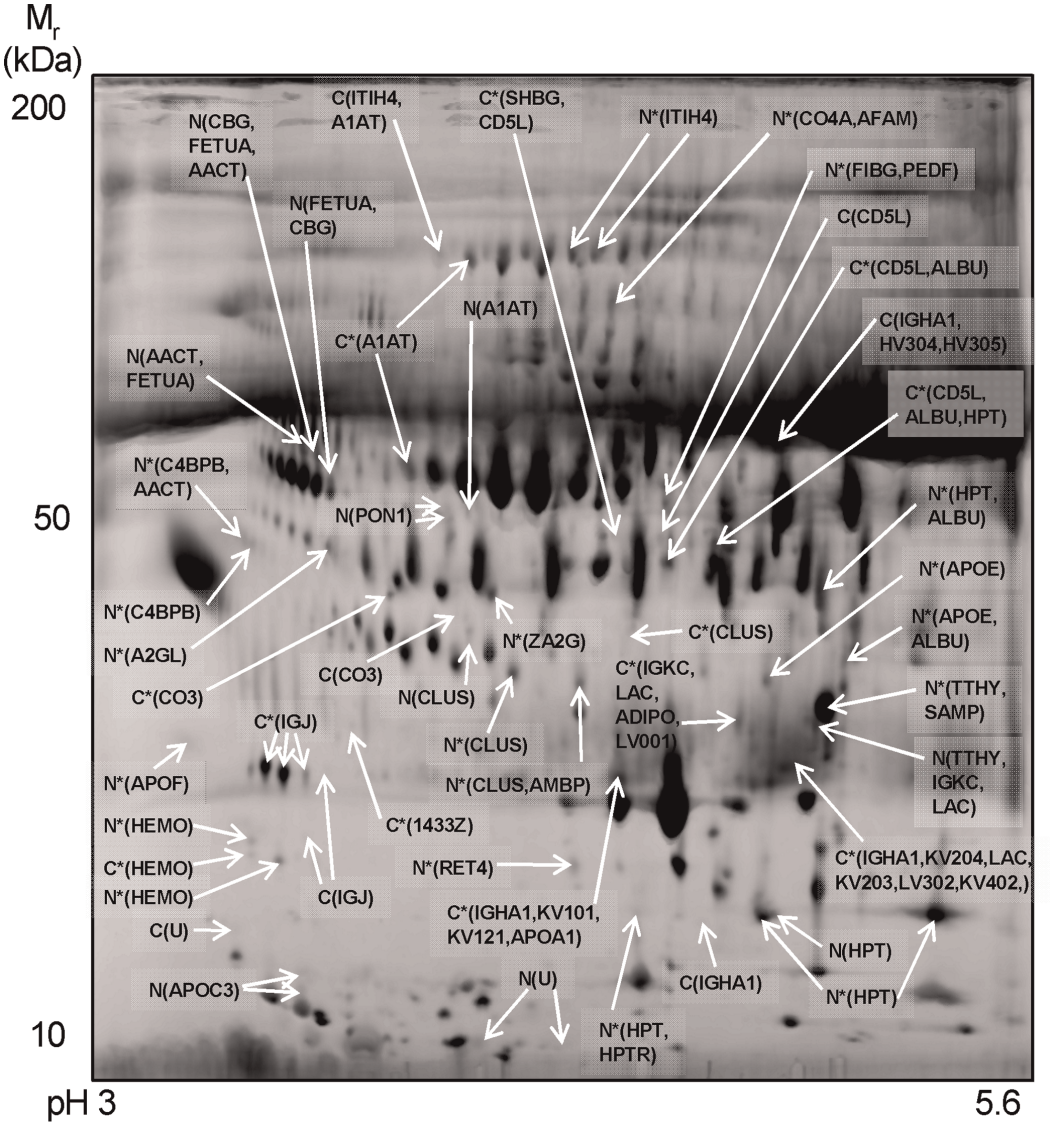
Synthetic 2-DE image representing all protein spots present in plasma samples in the comparison between normal healthy controls and cirrhosis patients. Gels were run using pH 3–5.6 nonlinear immobilized pH gradient DryStrips with 9–16% (w/v) SDS-PAGE gradient gels and were stained using the fluorescent dye OGT 1238. The synthetic image shown was created using accurate spot matching as previously described [19]. Differentially expressed features are indicated by arrows and the Swiss-Prot entry names are shown in parentheses. The names of selected proteins are shown in Table 2 and a full list of all proteins shown on this image can be found in Table S2. N, feature present only in gels of plasma from normal healthy controls; C, feature present only in gels of plasma from cirrhosis patients; *, features present in gels of plasma from both normal healthy controls and cirrhosis patients but expressed to a higher extent in the group indicated. For complete gel figures, see Figure S1.

**Figure 2:**
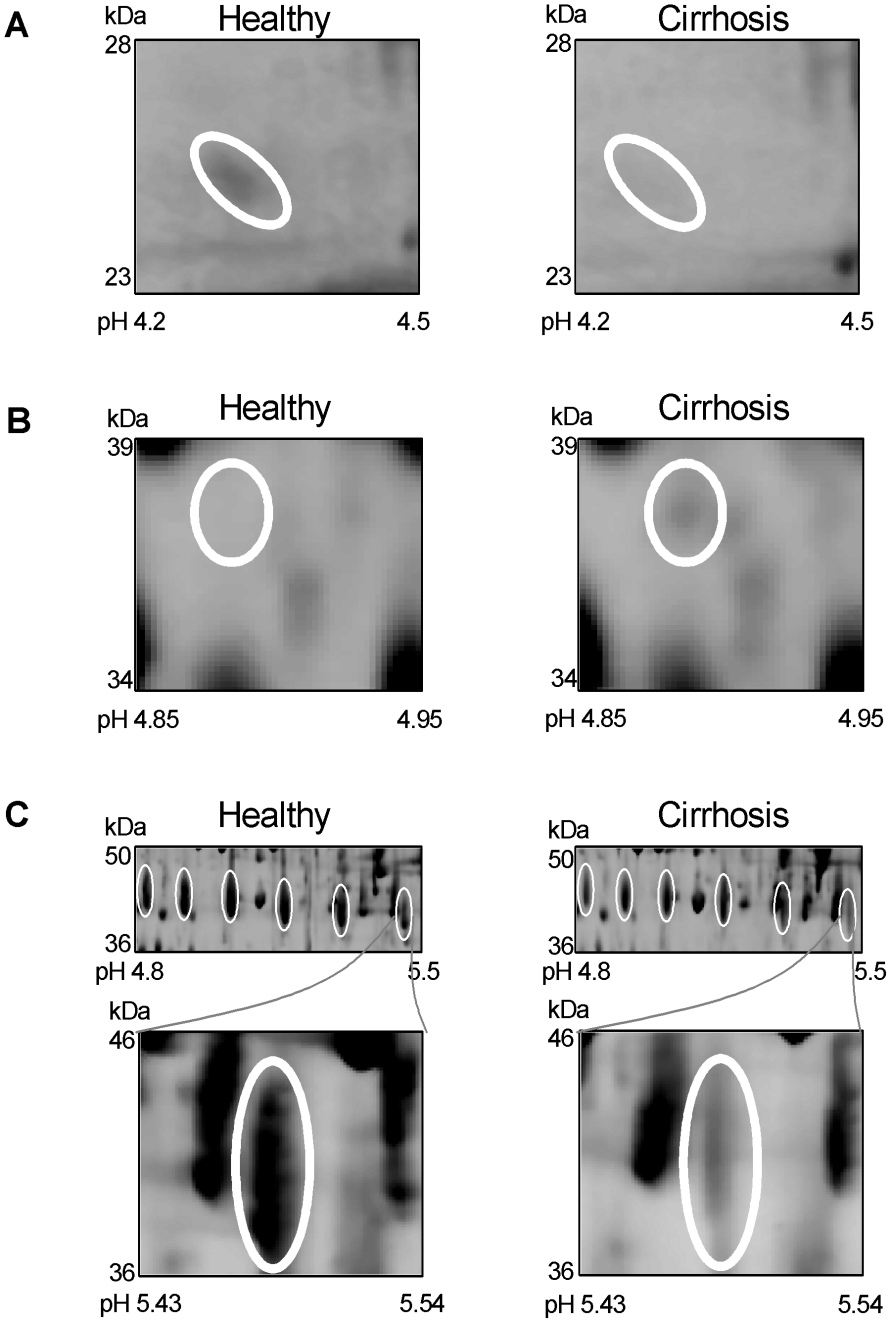
Magnified regions of the gels showing changes for selected potential novel fibrosis biomarkers. The relative position of the identified protein is circled. (A) LTIP is present in normal plasma but decreased in plasma from cirrhotic patients; (B) Zinc-alpha-2-glycoprotein is present in normal plasma and decreased in plasma from cirrhotic patients; (C) Decreased feature of beta haptoglobin at pH 5.46–5.49. The top panel shows evenly spaced array of beta haptoglobin spots showing no significant difference between normal plasma and plasma from cirrhotic patients. The bottom panel shows zoomed image of the beta haptoglobin spot observed at approximately pH 5.46–5.49 which is present in normal plasma and decreased in plasma from cirrhotic patients; (D) Complement C3dg is absent in normal plasma but present in plasma from cirrhotic patients.

A synthetic gel image representative of all features in the differential analysis comparing samples from all control and cirrhosis patients is shown in Figure 1. Original gel images for all ten gels are shown in Figure S1. Zoomed images of the gel regions depicting differential expression for selected novel candidate fibrosis biomarkers are shown in Figure 2. The image analysis software and statistical analysis found 243 statistically significant differentially expressed features of which 57 were considered suitable after validating by visualizing the differentially expressed features across all gels in a montage format. These 57 features were excised, digested with trypsin and analysed by LC-MSMS. Since some features contained more than one protein, among the 57 features a total of 85 proteins were identified and are listed in Table S2. For proteins identified with one peptide, the MSMS spectra are shown in Figure S2. Many of the proteins were identified as the same protein at different locations on the gels and so among these 85 proteins we identified 43 candidate biomarkers for hepatic fibrosis which are shown in Table S2. This table shows that in many cases the same protein spot contained more than one protein. The protein with the highest protein score has the greatest abundance and is therefore more likely to be the differentially expressed biomarker although the other proteins with lower protein score should not be ruled out. Among the 43 candidate biomarkers, 29 were top scoring proteins of which 20 were novel blood markers for fibrosis and not seen in our earlier study [13]. Table 2 shows a summary of all these 20 proteins with their function.

This analysis showed that expression of lipid transfer inhibitor protein, an isoform of beta haptoglobin at pH 5.46–5.49, haptoglobin-related protein, apolipoprotein C-III, apolipoprotein E, C4b-binding protein beta chain, retinol-binding protein 4, afamin, alpha-2-HS-glycoprotein, corticosteroid-binding globulin, leucine-rich alpha-2-glycoprotein and fibrinogen gamma chain was decreased in cirrhotic plasma, whereas expression of intact complement C3dg, immunoglobulin J chain, sex hormone-binding globulin, 14-3-3 protein zeta/delta and adiponectin increased. Features containing the glycoproteins alpha-1-antitrypsin, hemopexin and apolipoprotein J were increased and decreased at different locations on the gels suggesting potential post-translational modification of these proteins. In addition to these novel candidate markers for fibrosis, we also identified proteins already seen in our previous study [13] (decrease in albumin, α1 antichymotrypsin, complement C4, inter-α-trypsin inhibitor heavy chain H4, paraoxonase/arylesterase 1, zinc-alpha-2-glycoprotein and the elevation in immunoglobulin chains and CD5L).

### Haptoglobin and its Isoform at pH 5.46–5.49 as a Novel Fibrosis Marker

Haptoglobin was chosen for further peptide sequence analysis since an isoform of its beta chain decreased more consistently than the alpha chain of haptoglobin and the other beta haptoglobin isoforms. The decrease in haptoglobin in cirrhosis was observed at approximately 17 kDa and 40 kDa (Figure 1 and Table S2) and the identified peptides in Figure S3A show that the features at these molecular weights correspond to the alpha chain and glycosylated beta chain, respectively. The beta chain of haptoglobin was seen as an array of evenly spaced features between pH 4.7 and 5.5 (Figure 2C upper panel). The alpha and beta chains did not consistently decrease in cirrhosis (Figure 2C upper panel and Figure S3/S3C/S3DB). However, one isoform for beta haptoglobin at approximately pH 5.46–5.49 decreased more consistently than the alpha chain of haptoglobin and the other beta haptoglobin isoforms (Figure 2C and Figure S3E). The pH of this isoform was determined by calibrating all gels using landmarks with known pH and molecular weights.

**Table 2:** Differentially expressed proteins identified in the analysis between healthy control and cirrhotic plasma samples.

Spot change		Protein (Swiss-Prot entry name)	Function
Decreased in cirrhosis	⇓	Apolipoprotein C-III (APOC3)	Inhibits lipoprotein and hepatic lipases
	⇓	Corticosteroid-binding globulin (CBG)	Blood transport protein
	⇓	Alpha-2-HS-glycoprotein (FETUA)	Promotes endocytosis and possesses opsonic properties
	↓	Lipid transfer inhibitor protein LTIP (APOF)	LDL association. Inhibits cholesteryl ester transfer protein activity and regulator of cholesterol transport
	↓	β haptoglobin pH 5.46–5.49 (HPT)	Combines with free plasma hemoglobin, preventing loss of iron
	↓	Haptoglobin-related protein (HPTR)	Haptoglobin-related protein
	↓	Retinol-binding protein 4 (RET4)	Delivers retinol from liver to peripheral tissues. Prevents loss of transthyretin
	↓	Fibrinogen gamma chain (FIBG)	Yields monomers that polymerize into fibrin. Platelet aggregation cofactor
	↓	Leucine-rich alpha-2-glycoprotein (A2GL)	Role in protein-protein interactions, signal transduction, cell adhesion and development
	↓	Afamin (AFAM)	Possible role in the transport of yet unknown ligand
	↓	C4b-binding protein β chain (C4BPB)	Role in classical complement pathway
	↓	Apolipoprotein E (APOE)	Role in binding, internalisation and catabolism of lipoprotein particles
Post-translationally modified	⇓ ↓ ↑	Apolipoprotein J (CLUS)	Binds to cells, membranes and hydrophobic proteins
	⇓ ⇑ ↑	Alpha-1-antitrypsin (A1AT)	Serine protease inhibitor. Targets elastase, plasmin and thrombin
	↓ ↑	Hemopexin (HEMO)	Binds and transports heme to the liver
Increased in cirrhosis	↑	Adiponectin (ADIPO)	Involved in fat metabolism control
	↑	Sex hormone-binding globulin (SHBG)	Androgen transport protein
	↑	14-3-3 protein zeta/delta (1433Z)	Adapter protein implicated in signalling pathway regulation
	⇑ ↑	Complement C3dg (CO3)	Role in complement system
	⇑ ↑	Immunoglobulin J chain (IGJ)	Links two monomer units of IgM or IgA

Proteins shown were differentially expressed by 2-fold or more when comparing control and cirrhotic plasma gels.

⇓, feature present only in gels with plasma from normal healthy controls; ⇑, feature present only in gels with plasma from cirrhotic patients; ↓, feature present in both healthy and cirrhotic plasma but expressed to a higher extent in healthy plasma, ↑, feature present in both healthy and cirrhotic plasma but expressed to a higher extent in cirrhotic plasma.

Only novel markers of fibrosis are listed which were not seen in our previous 2-DE study. For a full list of all proteins identified, see Table S2.

### Complement C3dg Increases and Thioester Cleaved Complement C3 Decreases in Cirrhosis

**Figure 3:**
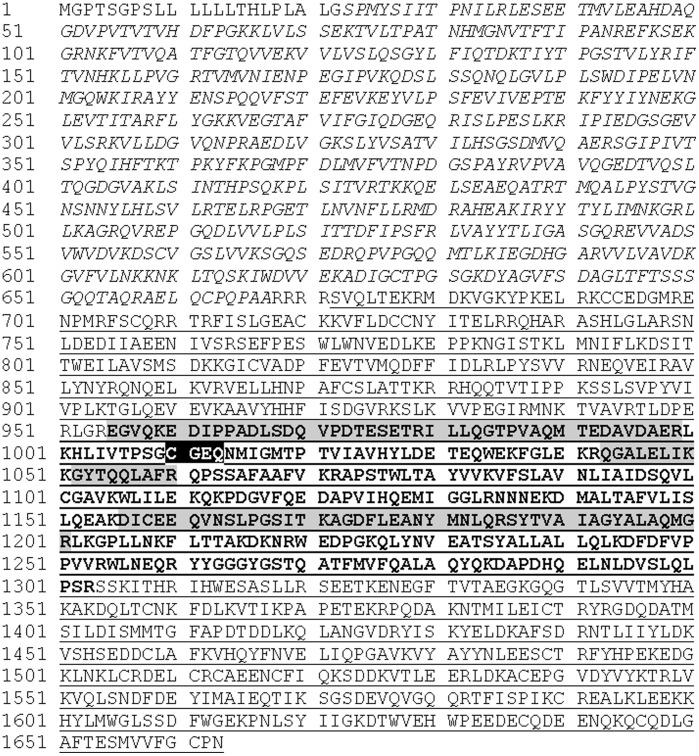
Uncleaved C3dg is elevated in hepatic cirrhosis. Using pH 3–5.6 gels, complement C3 was identified in a feature at approximately pH 4.9, MWt 38 kDa, only in gels for cirrhotic plasma. The full length sequence of complement C3 is shown with the alpha chain underlined, beta chain in italics, C3dg in bold and identified peptides highlighted in grey. Highlighted in black is the thioester site which is known to be cleaved by the fibrinolytic enzyme plasmin.

**Figure 4:**
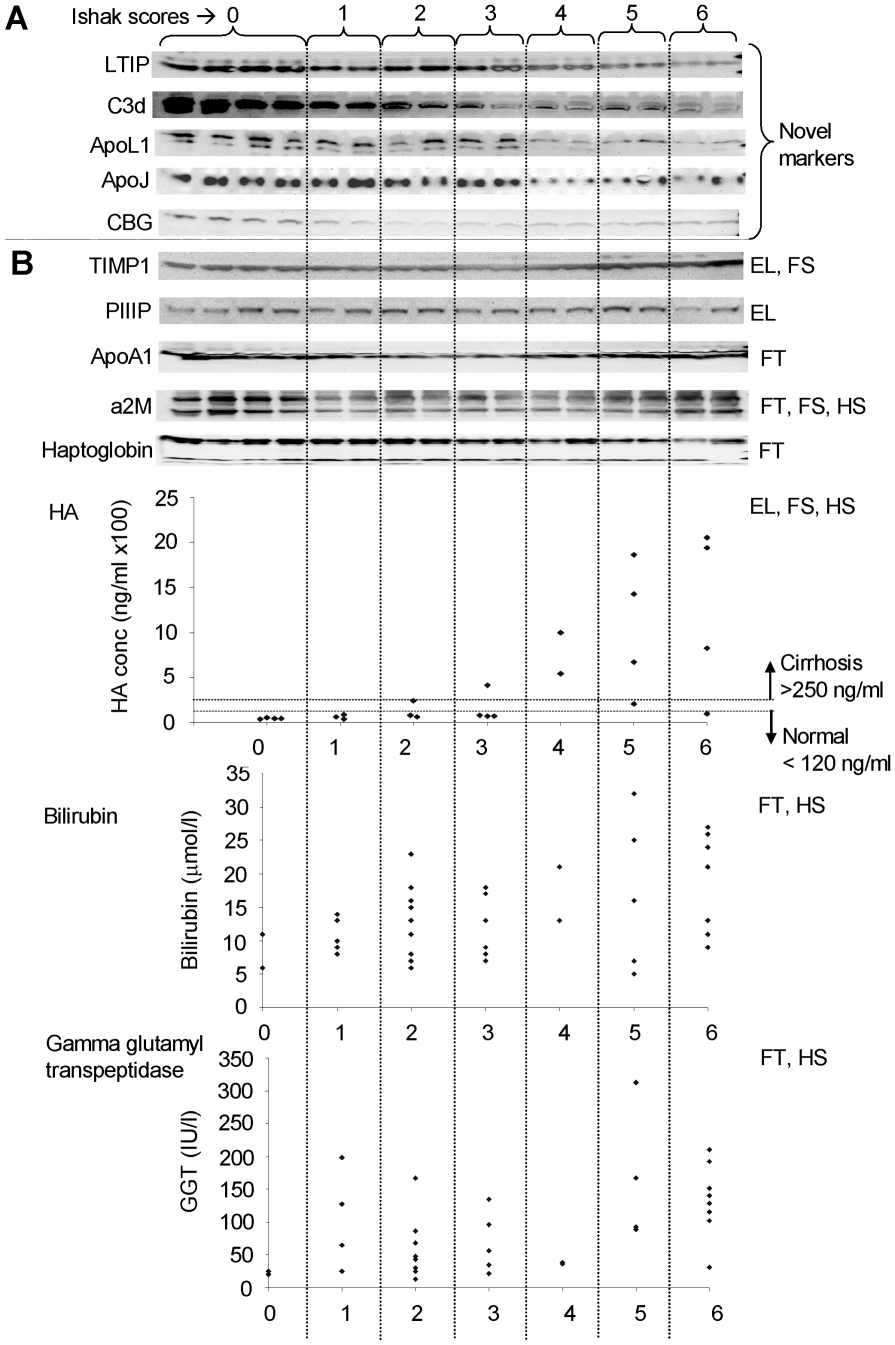
Validation of the novel fibrosis markers by Western blotting indicates that they are promising compared to the markers in ELF test, FibroTest, Hepascore and FIBROSpect. The novel markers of fibrosis were validated alongside the markers for the ELF test, FibroTest, Hepascore and FIBROSpect using plasma samples from individuals in each of the seven Ishak stages of hepatic scarring as indicated at the top of the figure. (A) Western blots of our novel markers of fibrosis: LTIP, complement C3d, apolipoprotein L1 (ApoL1), apolipoprotein J (ApoJ), corticosteroid-binding globulin (CBG); (B) ELF test, FibroTest, Hepascore and FIBROSpect markers. Western blots of TIMP-1, PIIIP, apolipoprotein A1 (Apo A1), alpha 2 macroglobulin (a2M) and haptoglobin, ELISA data for hyaluronic acid (HA) and levels of bilirubin and gamma glutamyltranspeptidase. For hyaluronic acid, normal individuals are recognised to have hyaluronic acid below 120 ng/ml and cirrhotic patients above 250 ng/ml as indicated with the dashed lines. The two letter codes indicate if the marker is used in ELF test (EL), FibroTest (FT), Hepascore (HS) or FIBROSpect (FS).

**Figure 5:**
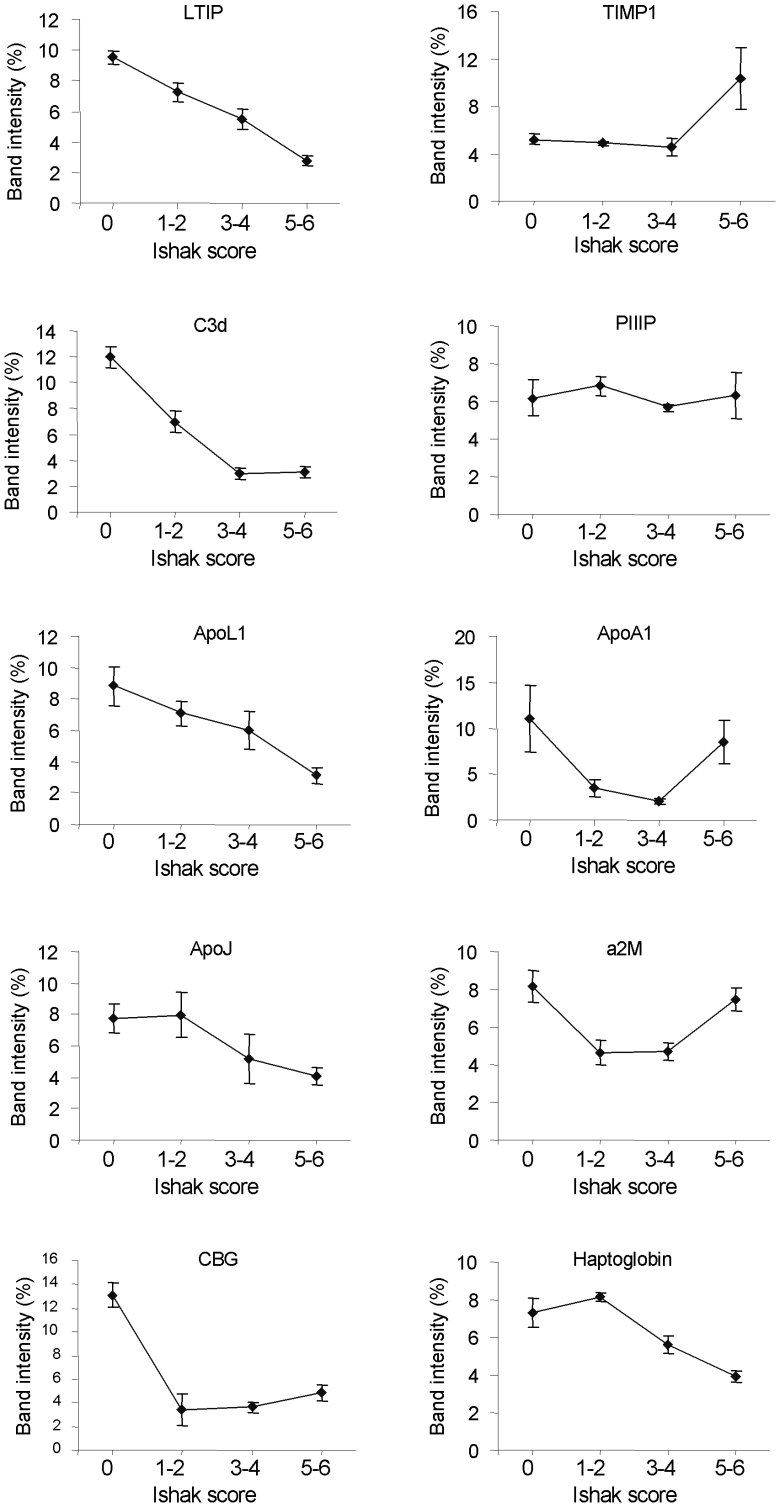
Western blot band densitometry. The five plots on the left show densitometry data for our five markers; from top to bottom: LTIP, complement C3d, apolipoprotein L1, apolipoprotein J and corticosteroid-binding globulin. The five plots on the right show densitometry data for all the markers that were blotted for in the ELF test (TIMP1 and PIIIP), FibroTest (apolipoprotein A1, alpha 2 macroglobulin and haptoglobin), Hepascore (alpha 2 macroglobulin) and FIBROSpect (TIMP-1, and alpha 2 macroglobulin). Each point represents the average band intensity for four patient samples. Error bars show +/− standard error.

Complement C3 has several cleavage products within its sequence and was chosen for further peptide sequence analysis to identify which cleavage product was higher in cirrhotic patient plasma. A fragment of complement C3 increased in cirrhosis and was observed on the 2-DE gel at 38 kDa with an approximate isoelectric point of pI 4.9 (Figure 2B). The peptide sequences identified by mass spectrometry are shown in Figure 3 and span from amino acids 955 to 1201. The amino acids for complement C3dg span from 955 to 1303 and its theoretical molecular weight and isoelectric point (39 kDa and pI 5) are in line with the observed gel feature indicating that the fragment of complement C3 in the feature is complement C3dg. Western blots using anti-complement C3d antibodies showed one band decreasing with increasing fibrosis stage (Figures 4 and 5), which is possibly thioester cleaved Complement C3 containing C3d.

### Biomarker Validation by Western Blotting

The novel candidate markers of fibrosis identified in this study and in our previous study [13] were validated by Western blotting using plasma from patients across all Ishak fibrosis scores. The same patient plasma samples were blotted for the protein markers in the FibroTest, ELF test, Hepascore and FIBROSpect and were compared to our novel fibrosis markers. Figures 4 and 5 show our top five markers which appear to be most reliably changing in expression across the Ishak fibrosis scores: lipid transfer inhibitor protein (LTIP), complement C3d, apolipoprotein J, corticosteroid-binding globulin and finally apolipoprotein L1 which we identified in our previous study [13]. These five markers are different from the markers used in FibroTest, ELF test, Hepascore, FIBROSpect and other hepatic fibrosis markers. LTIP and complement C3d appeared to be the most superior markers showing clear expression differences between neighbouring stages. Both apolipoprotein L1 and apolipoprotein J showed a clear change in expression between the early stages of fibrosis (up to Ishak stage 3) and more advanced hepatic fibrosis (Ishak stages 4–6). Corticosteroid-binding globulin was high in healthy controls and consistently lower in Ishak stages 1–6. Figure S4 shows Western blots for the other novel markers of fibrosis we identified (afamin, adiponectin, IgJ, hemopexin, 14-3-3zeta, apolipoprotein E, apolipoprotein C-III) as well as other markers we identified in a previous study [13] (beta 2 glycoprotein-I, inter-alpha-trypsin inhibitor heavy chain H4, CD5L and zinc-alpha-2-glycoprotein). Although these markers were identified to be significantly changing when comparing plasma and serum of healthy and cirrhotic individuals by 2-DE, they did not appear to show a reliable trend when analysing all Ishak stages by Western blotting. Figure 4 shows Western blot, ELISA and liver function test data for all the markers used in ELF test, FibroTest, Hepascore and FIBROSpect (TIMP-1, PIIIP, hyaluronic acid, haptoglobin, alpha 2 macroglobulin, apolipoprotein A1, total bilirubin and gamma glutamyltranspeptidase) using all Ishak fibrosis stages. The data in this figure and Figure 5 show that our top five markers show a consistent decrease with increasing fibrosis stage.

## Discussion

Currently the primary tool for diagnosing and assessing hepatic fibrosis is by liver biopsy and a less invasive and reliable biological marker is needed. In this study we identified 44 candidate biomarkers for hepatic fibrosis of which 20 are novel blood markers of fibrosis and not seen in our earlier study [13]. The novel markers of fibrosis were validated by Western blotting with a range of fibrosis scores. This validation helped us to find five promising biomarkers, LTIP, complement C3d, apolipoprotein J, corticosteroid-binding globulin and apolipoprotein L1. All of these markers looked very promising when compared to the markers in FibroTest, ELF test, Hepascore and FIBROSpect.

We have previously applied a proteomics approach using 2-DE over the wide pH 3–10 range to identify candidate fibrosis biomarkers [13]. Although there is the limitation of a narrow dynamic range when using gel-based proteomics there is the advantage of detecting post-translational modifications and according to a recent review [21], we have discovered more secreted human biomarkers in liver fibrosis than any other proteomics study which uses either mass spectrometry or gel-based approaches. In the study presented here, we have increased our panel of biomarkers for hepatic fibrosis and successfully identified more potential fibrosis biomarkers than our earlier proteomics study. The reason for our success in finding several more candidate markers is because we have used a new proteomics method that is more sensitive and helps to see low abundance biomarkers. We focused on the narrow pH 3–5.6 range of the plasma proteome using 2-DE. This range was chosen since it is outside the range of highly abundant albumin, transferrin and immunoglobulins and therefore this pH range allows a greater amount of plasma protein to be loaded onto the gels compared to our previous pH 3–10 study. The narrow pH range with the higher load also enhances representation of low abundance features. An alternative approach would be to deplete these highly abundant proteins prior to 2-DE. Albumin has been depleted in an earlier 2-DE fibrosis marker study by White and coworkers which has revealed the same biomarkers as identified in our previous study, but also apolipoprotein AIV [14] which was not observed in our previous or current studies. This highlights an advantage of using depletion compared to our approach and we are currently looking into immunodepletion of our samples. However our narrow pH range approach helped to identify other novel markers of fibrosis which were not observed by White and coworkers when using depletion, and all of these were different to the proteins used in FibroTest, ELF test, Hepascore and FIBROSpect and other established fibrosis tests. Also the P100 tubes used to collect the blood samples enhance preservation and recovery of proteins which may have helped to identify the novel biomarkers of fibrosis.

We confirmed some of the expression changes identified in our previous study and the new results agree with our previous findings. For example, although we had previously mentioned that zinc-alpha-2-glycoprotein and paraoxonase/arylesterase 1 could be decreasing in cirrhosis, these proteins were identified in a feature containing another protein, haptoglobin, which was identified with higher protein score and thus we were uncertain if these proteins could be candidate biomarkers. The increased separation used in this study clearly resolves differentially expressed features where only zinc-alpha-2-glycoprotein and paraoxonase/arylesterase 1 were identified as novel markers of fibrosis.

It is unclear why the proteins we identified are markers for liver fibrosis. Below we discuss the potential involvement of our promising five novel biomarkers of hepatic fibrosis and the potential involvement of the following novel fibrosis biomarkers is shown in Table S3: apolipoprotein C-III, apolipoprotein E, hemopexin, alpha-1-antichymotrypsin (gene SERPINA3), alpha-1-antitrypsin (gene SERPINA1), C4b-binding protein beta chain. Information about the proteins, including amino acid sequence and sites of glycosylation, was derived from the ExPASy database (http://www.expasy.ch/).

We found LTIP to be decreased in cirrhosis and to be the most promising novel biomarker of fibrosis changing across the Ishak stages when validated by Western blotting, showing clear differences in expression between neighbouring stages. LTIP is a 29 kDa glyco- and apolipoprotein found in both LDL and HDL which can inhibit lipid transfer between lipoproteins. LTIP is known to inhibit cholesteryl ester transfer protein (CETP)-mediated cholesteryl ester and triglyceride transfer [22]. To our knowledge LTIP has never been identified as a biomarker for liver fibrosis or described in any virus system. An increase in CETP has already been described in primary biliary cirrhosis [23] which is consistent with the LTIP decrease observed in our study, making this glycoprotein an attractive novel biomarker candidate for hepatic scarring. The CETPs, which can be inhibited by LTIP, are functionally similar to microsomal triglyceride transfer proteins (MTP) which are also involved in triglyceride and cholesteryl ester transport, but between phospholipid surfaces. Since both MTP and CETP are involved in triglyceride and cholesteryl ester transport, inhibitors against either act as lipid-lowering agents and inhibitors against MTP can reduce CETP activity [24] although it is not known if CETP inhibitors like LTIP can affect MTP activity. MTP is essential for HCV production and is enriched in membrane vesicles in which the virus replication complex is located [25]. While it is tempting to speculate on an association between LTIP and HCV due to these relationships between HCV-MTP-CETP and LTIP-CETP, the decreased LTIP expression is more likely to be associated with hepatic scarring due to the previously reported increase in CETP in cirrhosis [23].

In addition to LTIP, other proteins in the apolipoprotein family were found to be differentially expressed: post-translational modification of apolipoprotein J and decreased expression of apolipoproteins C-III and E. Apolipoprotein J is a glycoprotein chaperone associated with elastic fibres in liver fibrosis and cirrhosis [26]. Another apolipoprotein, L1, was found to decrease in our previous study [13] indicating that several proteins in the apolipoprotein family appear to be related to hepatic fibrosis. We are currently investigating whether other apolipoproteins, which we have not identified due to low abundance or their gel location outside the pH 3–5.6 range investigated, could also serve as biomarkers. Western blots of both apolipoprotein L1 and apolipoprotein J showed a similar trend in expression with consistently high levels in the early stages of fibrosis (up to Ishak stage 3) and consistently lower levels in more advanced hepatic scarring (Ishak stages 4–6). These markers would therefore be beneficial in differentiating patients with mild fibrosis from those with more advanced fibrosis.

We found an isoform of beta haptoglobin at pH 5.46–5.49 to be a novel marker of fibrosis which decreases in liver fibrosis. This acute phase protein is a tetramer consisting of two 16 kDa alpha chains with no potential sites of glycosylation and two 27 kDa beta chains each of which has four potential sites of N-glycosylation [27]. The array of evenly spaced features for the beta chain (Figure 2) is due to differences in glycosylation for each feature [28]. Total haptoglobin is known to decrease in fibrosis and is presently used along with other proteins to diagnose liver fibrosis [5]. Our data shows that total haptoglobin is unreliable as a fibrosis marker since both alpha and beta chains do not consistently decrease in cirrhosis (Figure S3). Furthermore, the 2-DE profile for cirrhosis patient 5 looks different to the other gels since, unlike the other cirrhotic samples, this patient had very low levels of haptoglobin. Some cirrhotic patients have very low haptoglobin and the observation was also noted in our previous study [13]. Therefore this gel is a fair representation of the expression of this protein in some cirrhotic samples. It also illustrates that haptoglobin is not consistent and its levels can be considerably different among cirrhotic patients. The isoform of beta haptoglobin identified as a novel marker of fibrosis appears to be more reliable than currently used total haptoglobin [5]. This isoform of haptoglobin has glycans which are mainly biantennary, both mono- or disialylated with hardly any tri- or tetra-antennary/ sialylated structures and less sialic acid and more monosialylated structures than the other haptoglobin isoforms of lower pH [29]. The different glycosylation pattern on this isoform compared to the other isoforms is currently being investigated. Hemolytic stress in haptoglobin-hemopexin double-null mice, but not single knock-out mice, causes pronounced fibrosis [30] suggesting that both haptoglobin and hemopexin, which was also identified in this study, are important for protection from liver fibrosis.

Three of the proteins identified to be differentially expressed were members of the serpin protease inhibitor family: gene names SERPINA6, SERPINA3 and SERPINA1. Corticosteroid-binding globulin (gene SERPINA6) is decreased in cirrhosis compared to healthy controls and our Western blot data showed that this marker was high in healthy controls and generally lower in Ishak stages 1–6. HCV patients with Ishak stage 0 disease would also need to be analysed to see if corticosteroid-binding globulin is beneficial in determining early hepatic scarring.

A fragment of complement C3 was found to be increased in cirrhosis. Two other proteins involved in the complement cascade, complement C4 and C4b-binding protein beta chain, were found to decrease in cirrhosis. Opposing the increase in complement C3 seen here, we have previously shown a fragment of C3 decreasing in cirrhosis [13]. In our earlier study we show that this fragment decreasing in cirrhosis contains peptides corresponding to the α-chain of C3 preceding its thioester site (the location of this site is highlighted in Figure 3). The cleavage at the thioester site was thought to be caused by plasmin, an enzyme involved in fibrinolysis, and the theoretical pI and molecular weight of the cleaved fragment were in agreement with the observed feature on the gel. This led us to suspect that the decreased levels of the thioester cleaved fragment may indicate less plasmin-mediated cleavage of the complement C3 α-chain, a finding consistent with hepatic scarring. We now further support this hypothesis by showing that C3dg, which is not cleaved since peptides were identified on either side of the thioester site (Figure 3), is elevated in cirrhosis. Complement C3dg can be cleaved at this thioester site by plasmin into C3d and C3g. Western blots were performed using anti-complement C3d antibodies to detect fragments of Complement C3 where C3dg is intact (expected increase in cirrhosis) and fragments of Complement C3 containing C3d where plasmin has cleaved the thioester site (expected decrease in cirrhosis). Results showed that there was only one band decreasing with increasing fibrosis stage (Figure 4 and 5). The increase in Complement C3 where C3dg is intact may have not been observed since it may be below the level of detection and the 2-DE data confirms that C3dg is in very low abundance (Figure 2B). The fact that the increase in C3dg is not visible by Western blotting is an advantage since if it was observed it may mask the decrease in thioester cleaved Complement C3 containing C3d making it a less reliable biomarker. To our knowledge, this is the first time it has been indicated that there is decreased thioester cleavage of complement C3 in cirrhosis.

Potential interactions between all the differentially expressed proteins were analysed using the Ingenuity Pathways Analysis software. A network diagram showing potential interactions between the proteins is shown in Figure S5. Interestingly the software recognized that some of the proteins identified were related to transforming growth factor beta-1 (TGF beta1) which is the main fibrogenic cytokine in hepatic scarring [2] suggesting that these proteins are related to pathways involved in fibrogenesis. The Ingenuity Pathways Analysis software showed that the proteins identified were most closely related to canonical pathways involved in the acute phase response which is consistent with the hepatic scarring process since inflammation has an association with fibrogenesis.

Information about the patients used in this study is shown in Table S1. The 50 patients analysed in this study varied in age and sex. It is preferable if markers are not dependent on categories such as these and so we aimed to eliminate any group specific hits from the outset. The FibroTest and Hepascore are dependent on age and gender [5], [10] whereas the ELF test which initially used age was later found to be independent of this category [7], [31]. For the Western blots in Figure 4, plasma samples from both males and females were analysed in most of the 7 Ishak stages of fibrosis (stages 0, 1, 2, 3 and 5) except for stages 4 and 6 where samples were only available from males (Table 1). Since our samples were from patients mixed in gender for most of the stages, the Western blot data suggest that our markers do not rely on age or sex and this will need to be confirmed using a greater number of samples.

### Conclusions

This study shows how we used 2-DE gels with a narrow pH 3–5.6 range to identify candidate plasma biomarkers for liver fibrosis in hepatitis C patients. We identified 44 candidate biomarkers for hepatic fibrosis in HCV patients of which 20 were novel biomarkers of fibrosis and not seen earlier [13]. Western blot validation helped to find five promising biomarkers, which we are currently further validating, alongside the other markers we identified here and in our previous study, using dot blotting and ELISAs with a statistically relevant larger patient population. This will help us to determine the concentrations of each of our markers in plasma and their precision and performance. We are also looking into comparing markers found by gel-free mass spectrometry-based approaches with the markers found by our gel-based approaches. In addition our markers will be investigated in other cases of hepatic fibrosis (e.g HBV and alcohol-mediated), other chronic liver diseases and other scarring diseases (e.g. cardiac and skin fibrosis). The use of dot blotting and ELISAs should confirm that one or a combination of the top five proteins already identified can be incorporated into a clinical assay which can then be used to establish a scoring system to aid in the assessment of hepatic scarring. Such an assay would also aid in assessing fibrosis reduction during therapy which would help clinicians to monitor patient improvement during current therapy and also help in clinical trials where new anti-fibrotic drugs are to be investigated. Ultimately this assay would help clinicians to determine the severity of hepatic fibrosis and eliminate the need for invasive liver biopsies.

## Supporting Information

Figure S1 **2 mg of plasma from five healthy individuals (Normal 1–5) and five cirrhotic patients (Cirrhosis 1–5) were separated by 9–16% 2-DE using pH 3–5.6NL IPG strips.** Differentially expressed features along with their Swiss-Prot entry names are highlighted. N, feature present only in gels with plasma from normal healthy controls; C, feature present only in gels with plasma from cirrhotic patients; *, features present in both healthy and cirrhotic plasma but expressed to a higher extent in the group indicated.
(PPT)

Figure S2 **MSMS spectra for proteins identified by a single peptide.** All spectra were derived from Mascot. Peptide fragmentation patterns were generated using the observed singly charged y- or b-ions. Fragment ions minus H2O and NH3 were omitted.
(PDF)

Figure S3 **Haptoglobin expression analysis by 2-DE and Western blotting.** (A) A decrease in haptoglobin in cirrhosis was observed in gel features at approximately 17 kDa and 40 kDa. The full length sequence of haptoglobin is shown with the alpha chain underlined and beta chain in bold. Peptides identified in the features at 17 kDa are highlighted in light grey and for the feature at 40 kDa in dark grey. (B) Magnified regions of the 2D gels at approximately 40 kDa showing the array of beta haptoglobin spots (arrowed). (C) Magnified regions of the 2D gels at approximately 17 kDa showing the alpha haptoglobin spots (circled). (D)Western blot of haptoglobin using plasma from 5 healthy individuals and 10 cirrhotic patients. This confirms the data in figures C and D that there is no significant difference in the expression of both alpha and beta haptoglobin when comparing plasma from normal individuals with plasma from cirrhotic patients. (E) Upper panel  =  Magnified regions of the 2D gels at approximately 40 kDa showing an isoform of beta haptoglobin (circled) between pH 5.46–5.49 to the right of the beta haptoglobin array. Lower panel  =  Zoomed in image of the beta haptoglobin feature between pH 5.46–5.49 (circled).
(DOC)

Figure S4 **Western blot validation.** Four plasma samples from controls (Ishak score 0) and two plasma samples from patients in each of the six Ishak stages of hepatic scarring (stages 1–6) were run on 17-well SDS-PAGE gels. Separated plasma proteins were electroblotted onto nitrocellulose membranes and probed with the following primary antibodies: afamin, adiponectin, IgJ, hemopexin, 14-3-3zeta, apolipoprotein E (Apo E), apolipoprotein C3 (Apo C3), beta 2 glycoprotein-I (B2GPI), inter-alpha-trypsin inhibitor heavy chain H4 (ITIH4), CD5L and zinc-alpha-2-glycoprotein (ZAG). Bands were detected with ECL Plus.
(DOC)

Figure S5 **Ingenuity Pathway Analysis.** Differentially expressed proteins were analysed using the Ingenuity Pathway Analysis software. Potential protein interactions are shown. Identified proteins are coloured and labelled with their gene names as shown in Table S1. Potential interacting partners which were not identified in the 2-DE study are shown in white. Solid lines (green, red, white, pink) represent direct interactions, dashed lines (yellow, grey) represent indirect interactions. Arrows (white, yellow, red, pink) from one protein node to another indicates that the node acts on the other node. Lines without arrowheads (green) represent binding. Lines with a small perpendicular line at the end (grey) represent inhibition. Proteins identified by differential analysis are shown as coloured nodes whereas unidentified proteins are white.
(DOC)

Method S1 **Biomarker validation.**
(DOC)

Table S1 **Clinical details of all 50 plasma samples.** na = not analysed. * = biopsy not performed to indicate Ishak score 0, but these individuals all declared that they had no known pathology and all were fit and well at the time of sampling. Clinical blood measurements not taken at the time of sampling are preceded with the date to show how close this value was measured to the time of sampling.
(XLS)

Table S2 **Differentially expressed proteins identified in plasma samples of healthy controls versus cirrhotic patients.** Entries in blue indicate proteins which had the highest score within a protein spot. Among these, the protein names and entries in bold are novel and were not seen in our earlier study. [13] AN, Swiss-Prot accession number; N, feature present in plasma from healthy controls; C, feature present in plasma from cirrhosis patients. Fold change refers to proteins that were differentially expressed by 2-fold or more when comparing plasma gels from healthy controls with cirrhosis. The numerical values shown in parentheses for fold change indicate features that were present in both controls and cirrhosis but expressed to a higher extent in the indicated stage. For cases where no numerical value is shown for fold change, the feature was only present in the indicated stage. pI, isoelectric point on gel as determined by the image analysis software using calibrated landmarks; MWt, molecular weight on gel as determined by the image analysis software using calibrated landmarks. The number of MS/MS peptide matches, percentage sequence coverage and protein score were determined by the Mascot Daemon search engine. Protein functions have been adapted from the ExPASy website.
(DOC)

Table S3 **Potential involvement of a selection of the novel proteins in hepatic scarring.**
(DOC)
