# Supplementary material for: Discovery of Novel Biomarker Candidates for Liver Fibrosis in Hepatitis C Patients: A Preliminary Study
Source: PLoS One. 2012 Jun 26;7(6):e39603. doi: 10.1371/journal.pone.0039603 (PMC3383672; doi:10.1371/journal.pone.0039603)
Supplement: Figure S1 — 2 mg of plasma from five healthy individuals (Normal 1–5) and five cirrhotic patients (Cirrhosis 1–5) were separated by 9–16% 2-DE using pH 3–5.6NL IPG strips. Differentially expressed features along with their Swiss-Prot entry names are highlighted. N, feature present only in gels with plasma from normal healthy controls; C, feature present only in gels with plasma from cirrhotic patients; *, features present in both healthy and cirrhotic plasma but expressed to a higher extent in the group indicated. (PPT) [file pone.0039603.s001.ppt]

## Slide 1
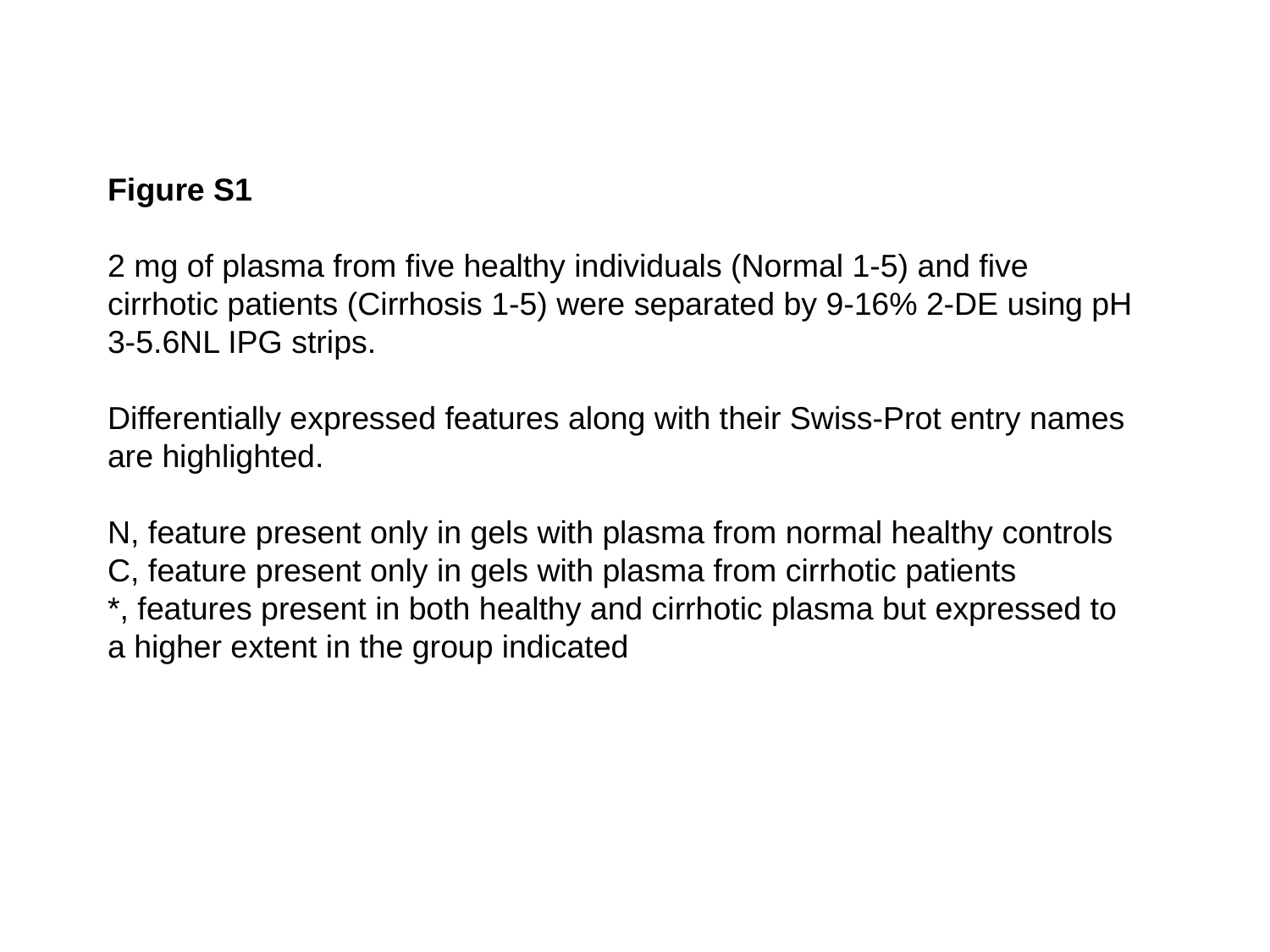

Figure S1
2 mg of plasma from five healthy individuals (Normal 1-5) and five cirrhotic patients (Cirrhosis 1-5) were separated by 9-16% 2-DE using pH 3-5.6NL IPG strips.
Differentially expressed features along with their Swiss-Prot entry names are highlighted.
N, feature present only in gels with plasma from normal healthy controls
C, feature present only in gels with plasma from cirrhotic patients
*, features present in both healthy and cirrhotic plasma but expressed to a higher extent in the group indicated

## Slide 2
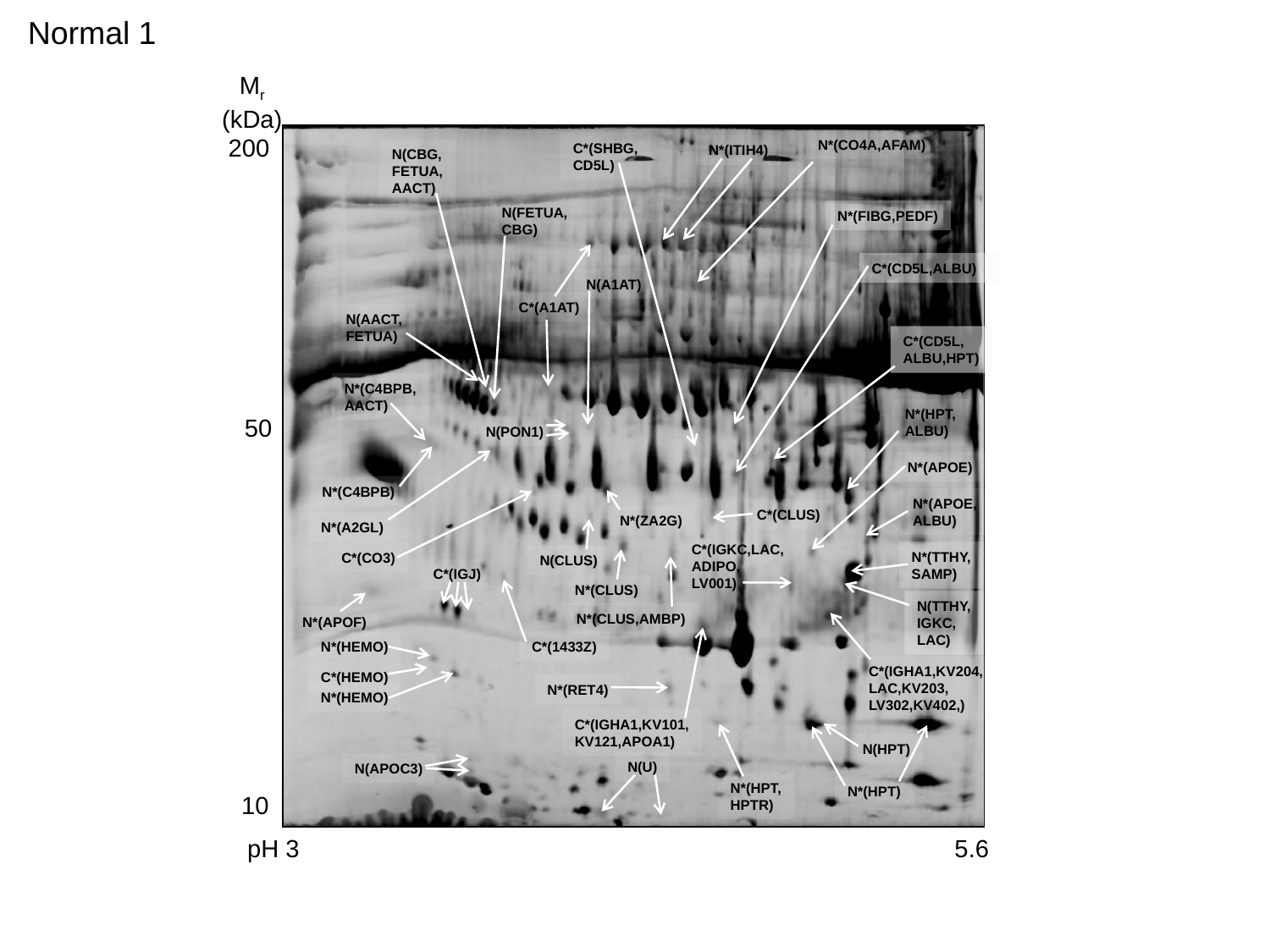

Normal 1
Mr
(kDa)
200
N*(CO4A,AFAM)
C*(SHBG,
CD5L)
N*(ITIH4)
N(CBG,
FETUA,
AACT)
N(FETUA,
CBG)
N*(FIBG,PEDF)
C*(CD5L,ALBU)
N(A1AT)
C*(A1AT)
N(AACT,
FETUA)
C*(CD5L,
ALBU,HPT)
N*(C4BPB,
AACT)
N*(HPT,
ALBU)
50
N(PON1)
N*(APOE)
N*(C4BPB)
N*(APOE,
ALBU)
C*(CLUS)
N*(ZA2G)
N*(A2GL)
C*(IGKC,LAC,
ADIPO,
LV001)
N*(TTHY,
SAMP)
C*(CO3)
N(CLUS)
C*(IGJ)
N*(CLUS)
N(TTHY,
IGKC,
LAC)
N*(CLUS,AMBP)
N*(APOF)
C*(1433Z)
N*(HEMO)
C*(IGHA1,KV204,
LAC,KV203,
LV302,KV402,)
C*(HEMO)
N*(RET4)
N*(HEMO)
C*(IGHA1,KV101,
KV121,APOA1)
N(HPT)
N(U)
N(APOC3)
N*(HPT,
HPTR)
N*(HPT)
10
pH 3 5.6

## Slide 3
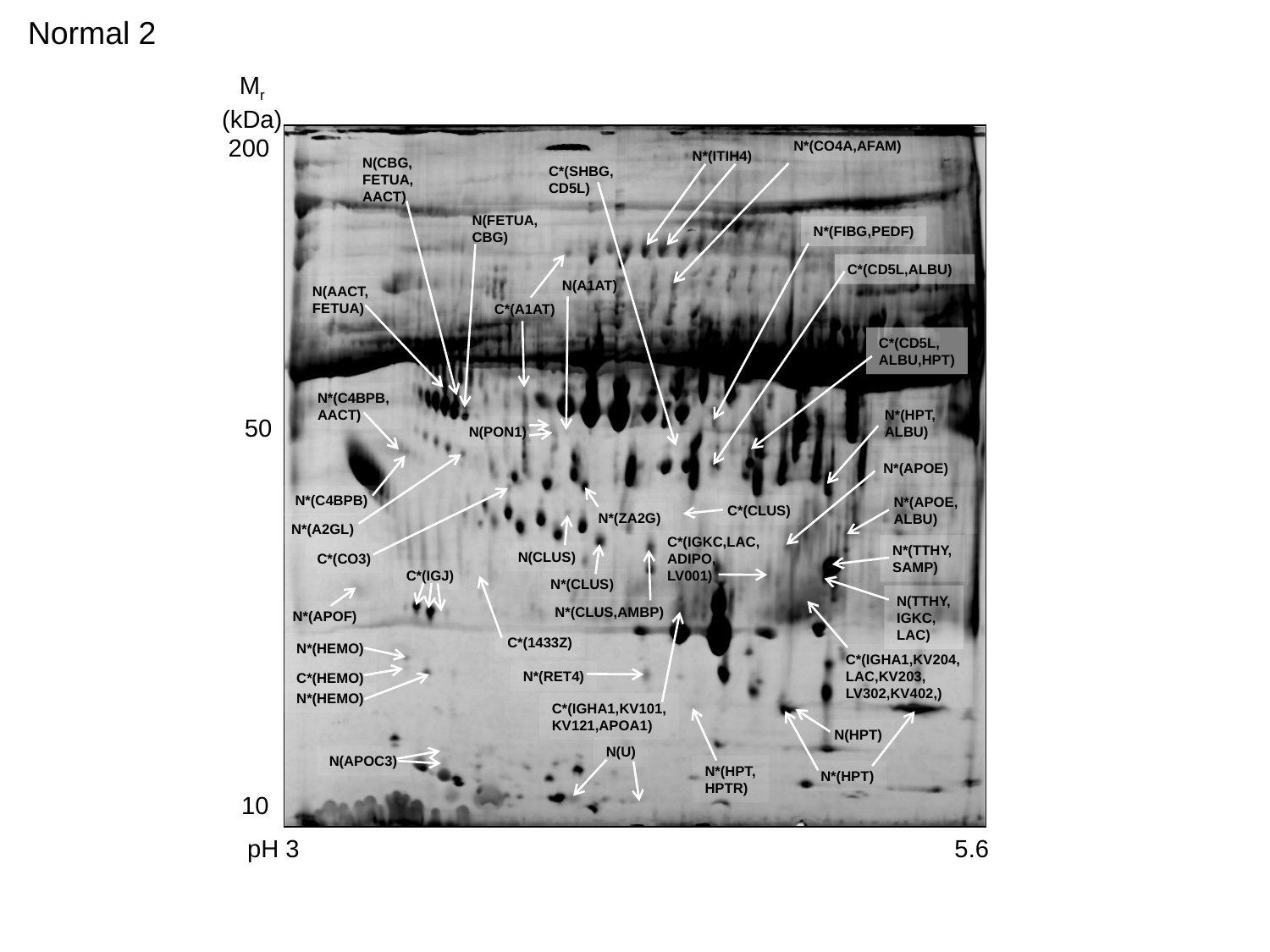

Normal 2
Mr
(kDa)
200
N*(CO4A,AFAM)
N*(ITIH4)
N(CBG,
FETUA,
AACT)
C*(SHBG,
CD5L)
N(FETUA,
CBG)
N*(FIBG,PEDF)
C*(CD5L,ALBU)
N(A1AT)
N(AACT,
FETUA)
C*(A1AT)
C*(CD5L,
ALBU,HPT)
N*(C4BPB,
AACT)
N*(HPT,
ALBU)
50
N(PON1)
N*(APOE)
N*(C4BPB)
N*(APOE,
ALBU)
C*(CLUS)
N*(ZA2G)
N*(A2GL)
C*(IGKC,LAC,
ADIPO,
LV001)
N*(TTHY,
SAMP)
N(CLUS)
C*(CO3)
C*(IGJ)
N*(CLUS)
N(TTHY,
IGKC,
LAC)
N*(CLUS,AMBP)
N*(APOF)
C*(1433Z)
N*(HEMO)
C*(IGHA1,KV204,
LAC,KV203,
LV302,KV402,)
N*(RET4)
C*(HEMO)
N*(HEMO)
C*(IGHA1,KV101,
KV121,APOA1)
N(HPT)
N(U)
N(APOC3)
N*(HPT,
HPTR)
N*(HPT)
10
pH 3 5.6

## Slide 4
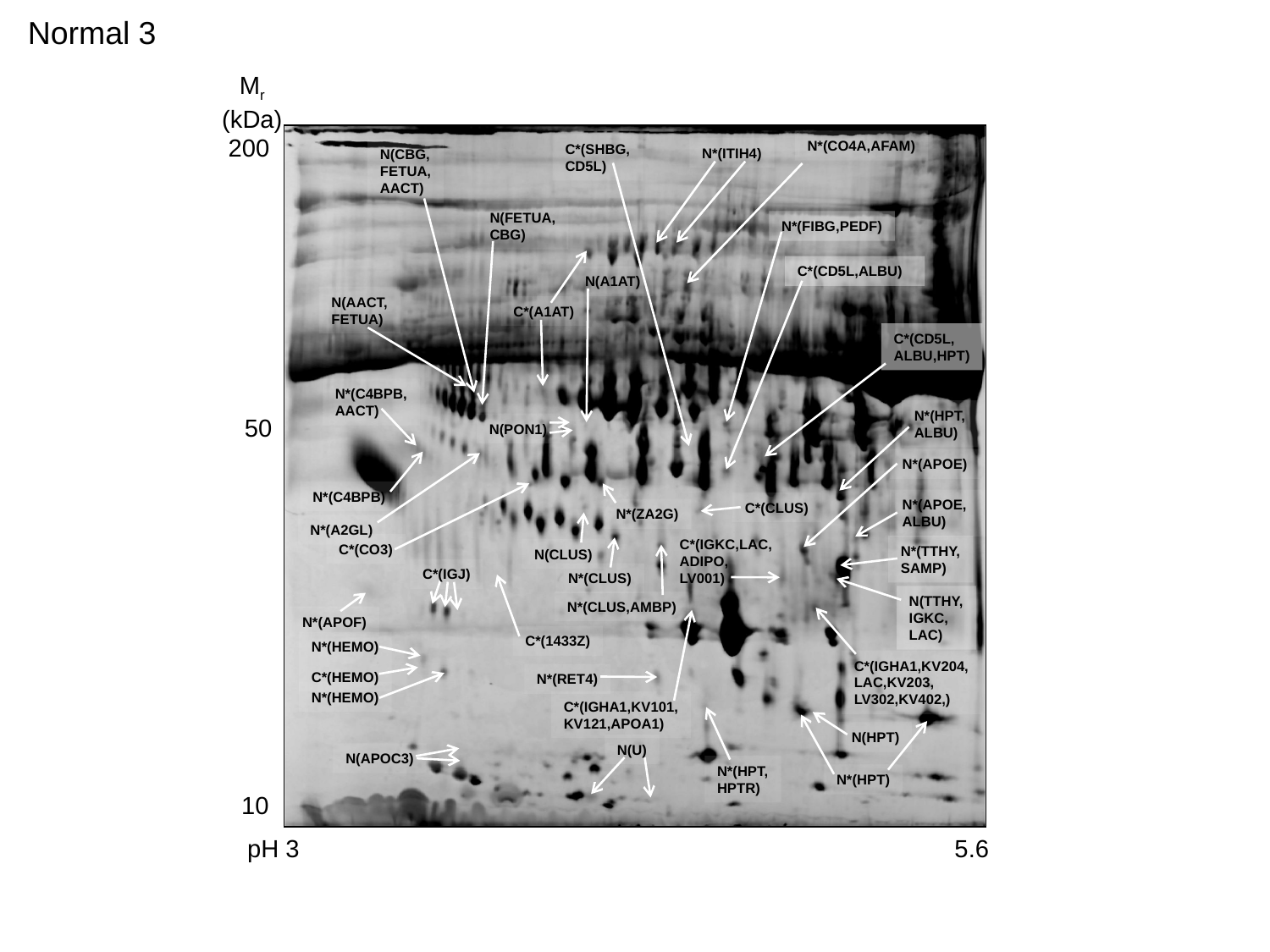

Normal 3
Mr
(kDa)
200
N*(CO4A,AFAM)
C*(SHBG,
CD5L)
N*(ITIH4)
N(CBG,
FETUA,
AACT)
N(FETUA,
CBG)
N*(FIBG,PEDF)
C*(CD5L,ALBU)
N(A1AT)
N(AACT,
FETUA)
C*(A1AT)
C*(CD5L,
ALBU,HPT)
N*(C4BPB,
AACT)
N*(HPT,
ALBU)
50
N(PON1)
N*(APOE)
N*(C4BPB)
N*(APOE,
ALBU)
C*(CLUS)
N*(ZA2G)
N*(A2GL)
C*(IGKC,LAC,
ADIPO,
LV001)
C*(CO3)
N*(TTHY,
SAMP)
N(CLUS)
C*(IGJ)
N*(CLUS)
N(TTHY,
IGKC,
LAC)
N*(CLUS,AMBP)
N*(APOF)
C*(1433Z)
N*(HEMO)
C*(IGHA1,KV204,
LAC,KV203,
LV302,KV402,)
C*(HEMO)
N*(RET4)
N*(HEMO)
C*(IGHA1,KV101,
KV121,APOA1)
N(HPT)
N(U)
N(APOC3)
N*(HPT,
HPTR)
N*(HPT)
10
pH 3 5.6

## Slide 5
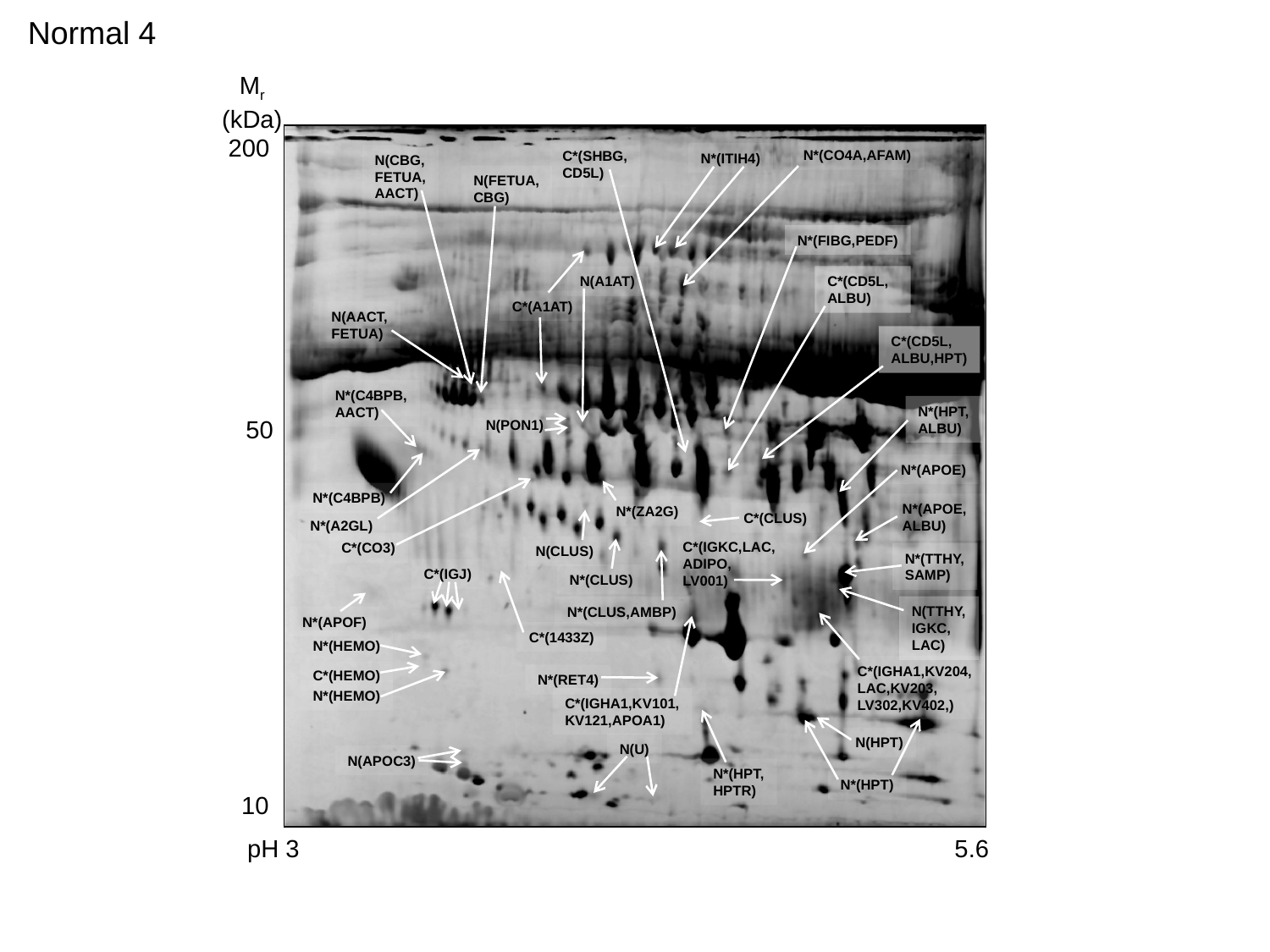

Normal 4
Mr
(kDa)
200
N*(CO4A,AFAM)
C*(SHBG,
CD5L)
N*(ITIH4)
N(CBG,
FETUA,
AACT)
N(FETUA,
CBG)
N*(FIBG,PEDF)
C*(CD5L,
ALBU)
N(A1AT)
C*(A1AT)
N(AACT,
FETUA)
C*(CD5L,
ALBU,HPT)
N*(C4BPB,
AACT)
N*(HPT,
ALBU)
50
N(PON1)
N*(APOE)
N*(C4BPB)
N*(APOE,
ALBU)
N*(ZA2G)
C*(CLUS)
N*(A2GL)
C*(IGKC,LAC,
ADIPO,
LV001)
C*(CO3)
N(CLUS)
N*(TTHY,
SAMP)
C*(IGJ)
N*(CLUS)
N(TTHY,
IGKC,
LAC)
N*(CLUS,AMBP)
N*(APOF)
C*(1433Z)
N*(HEMO)
C*(IGHA1,KV204,
LAC,KV203,
LV302,KV402,)
C*(HEMO)
N*(RET4)
N*(HEMO)
C*(IGHA1,KV101,
KV121,APOA1)
N(HPT)
N(U)
N(APOC3)
N*(HPT,
HPTR)
N*(HPT)
10
pH 3 5.6

## Slide 6
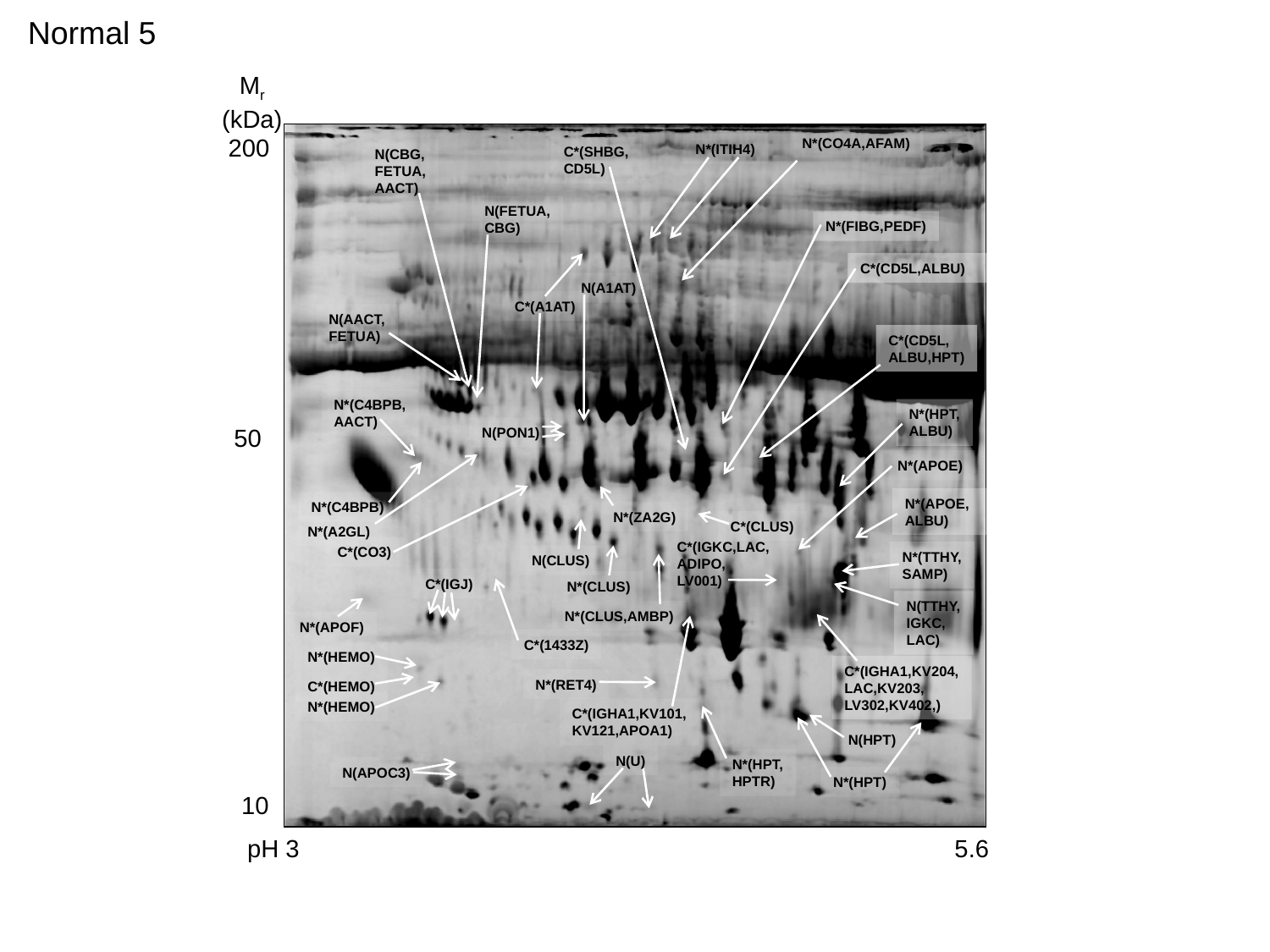

Normal 5
Mr
(kDa)
200
N*(CO4A,AFAM)
N*(ITIH4)
C*(SHBG,
CD5L)
N(CBG,
FETUA,
AACT)
N(FETUA,
CBG)
N*(FIBG,PEDF)
C*(CD5L,ALBU)
N(A1AT)
C*(A1AT)
N(AACT,
FETUA)
C*(CD5L,
ALBU,HPT)
N*(C4BPB,
AACT)
N*(HPT,
ALBU)
50
N(PON1)
N*(APOE)
N*(APOE,
ALBU)
N*(C4BPB)
N*(ZA2G)
C*(CLUS)
N*(A2GL)
C*(IGKC,LAC,
ADIPO,
LV001)
C*(CO3)
N*(TTHY,
SAMP)
N(CLUS)
C*(IGJ)
N*(CLUS)
N(TTHY,
IGKC,
LAC)
N*(CLUS,AMBP)
N*(APOF)
C*(1433Z)
N*(HEMO)
C*(IGHA1,KV204,
LAC,KV203,
LV302,KV402,)
N*(RET4)
C*(HEMO)
N*(HEMO)
C*(IGHA1,KV101,
KV121,APOA1)
N(HPT)
N(U)
N*(HPT,
HPTR)
N(APOC3)
N*(HPT)
10
pH 3 5.6

## Slide 7
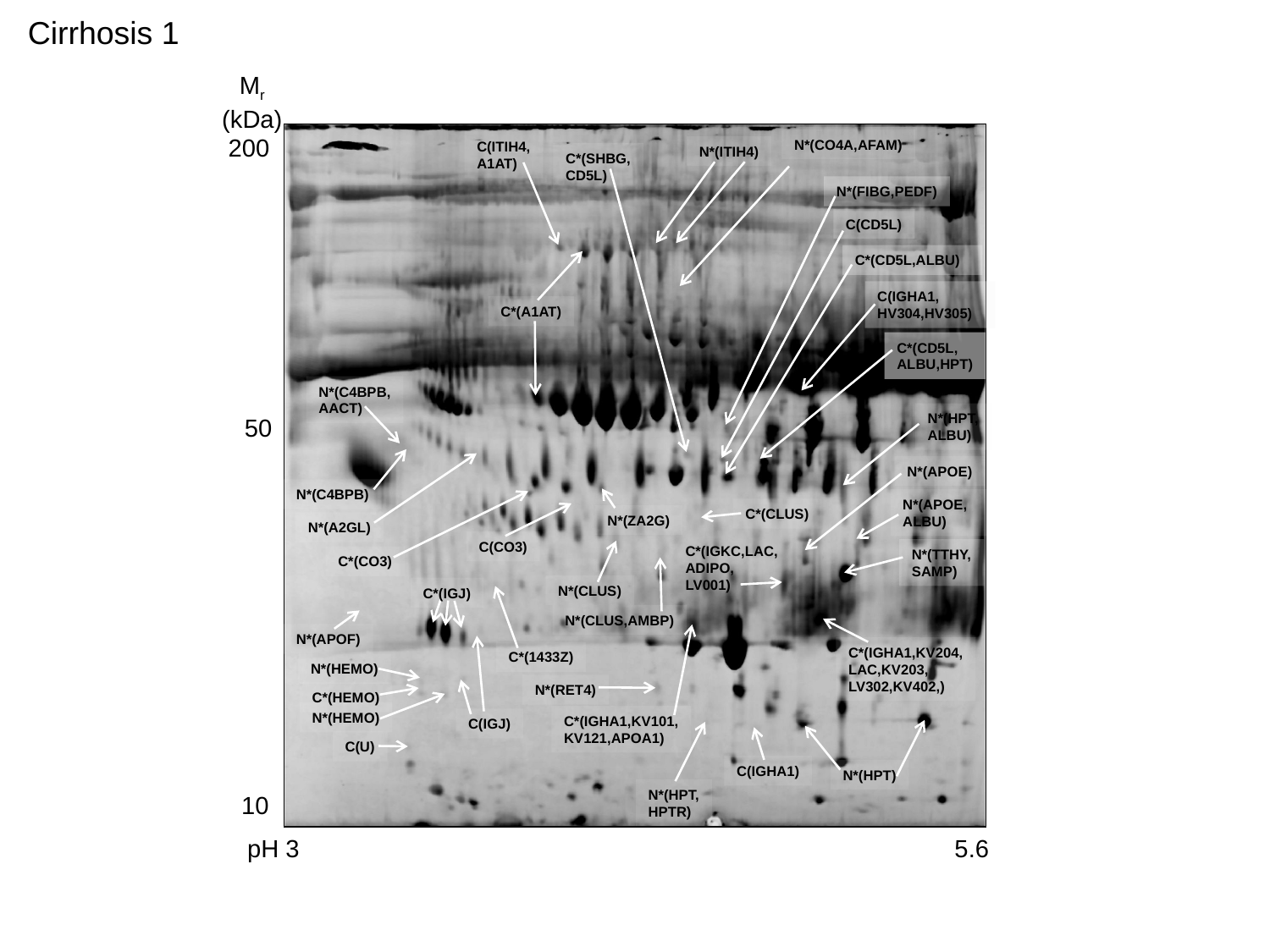

Cirrhosis 1
Mr
(kDa)
200
N*(CO4A,AFAM)
C(ITIH4,
A1AT)
N*(ITIH4)
C*(SHBG,
CD5L)
N*(FIBG,PEDF)
C(CD5L)
C*(CD5L,ALBU)
C(IGHA1,
HV304,HV305)
C*(A1AT)
C*(CD5L,
ALBU,HPT)
N*(C4BPB,
AACT)
N*(HPT,
ALBU)
50
N*(APOE)
N*(C4BPB)
N*(APOE,
ALBU)
C*(CLUS)
N*(ZA2G)
N*(A2GL)
C(CO3)
C*(IGKC,LAC,
ADIPO,
LV001)
N*(TTHY,
SAMP)
C*(CO3)
N*(CLUS)
C*(IGJ)
N*(CLUS,AMBP)
N*(APOF)
C*(IGHA1,KV204,
LAC,KV203,
LV302,KV402,)
C*(1433Z)
N*(HEMO)
N*(RET4)
C*(HEMO)
N*(HEMO)
C*(IGHA1,KV101,
KV121,APOA1)
C(IGJ)
C(U)
C(IGHA1)
N*(HPT)
N*(HPT,
HPTR)
10
pH 3 5.6

## Slide 8
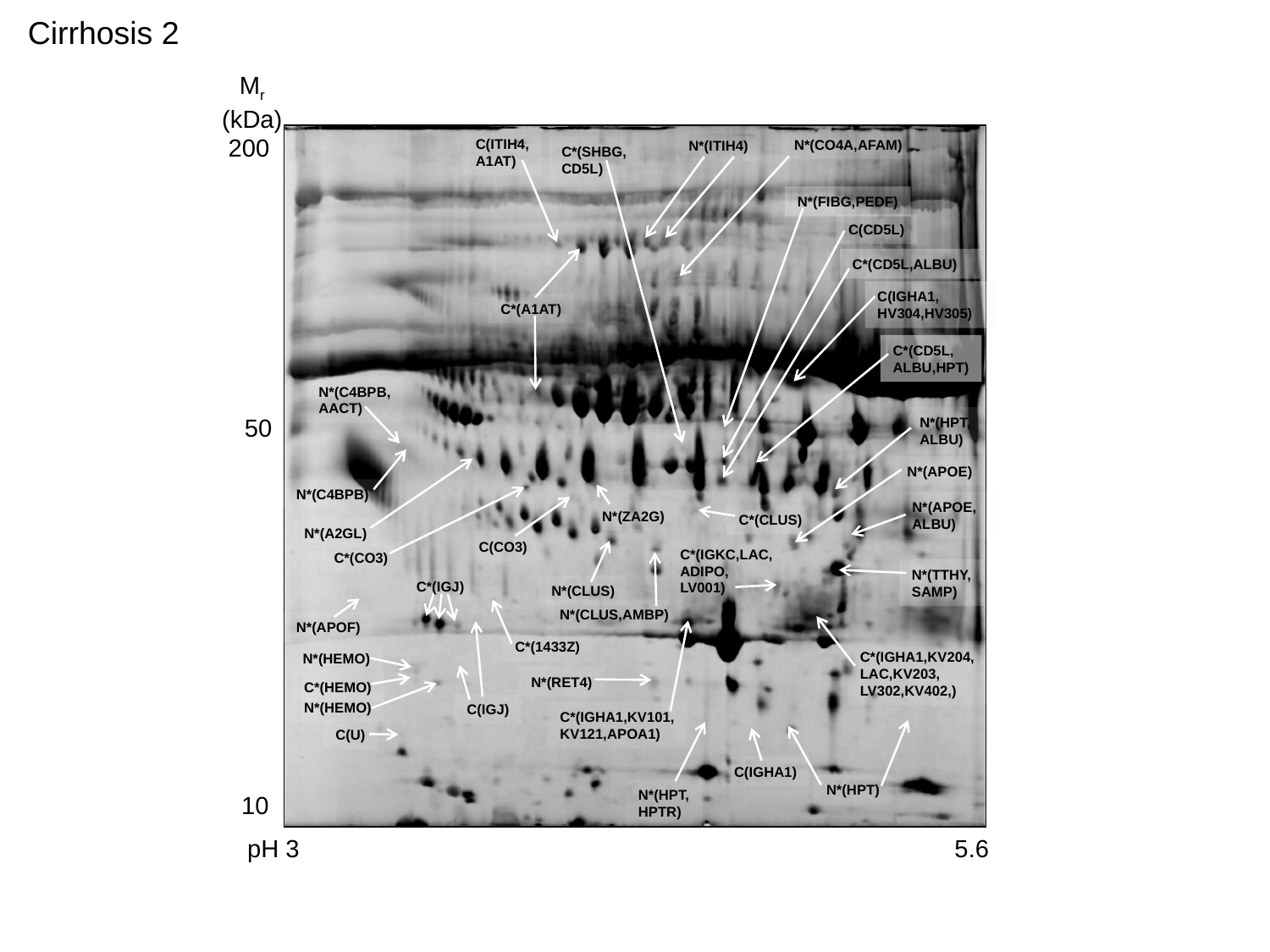

Cirrhosis 2
Mr
(kDa)
200
C(ITIH4,
A1AT)
N*(CO4A,AFAM)
N*(ITIH4)
C*(SHBG,
CD5L)
N*(FIBG,PEDF)
C(CD5L)
C*(CD5L,ALBU)
C(IGHA1,
HV304,HV305)
C*(A1AT)
C*(CD5L,
ALBU,HPT)
N*(C4BPB,
AACT)
50
N*(HPT,
ALBU)
N*(APOE)
N*(C4BPB)
N*(APOE,
ALBU)
N*(ZA2G)
C*(CLUS)
N*(A2GL)
C(CO3)
C*(IGKC,LAC,
ADIPO,
LV001)
C*(CO3)
N*(TTHY,
SAMP)
C*(IGJ)
N*(CLUS)
N*(CLUS,AMBP)
N*(APOF)
C*(1433Z)
C*(IGHA1,KV204,
LAC,KV203,
LV302,KV402,)
N*(HEMO)
N*(RET4)
C*(HEMO)
N*(HEMO)
C(IGJ)
C*(IGHA1,KV101,
KV121,APOA1)
C(U)
C(IGHA1)
N*(HPT)
N*(HPT,
HPTR)
10
pH 3 5.6

## Slide 9
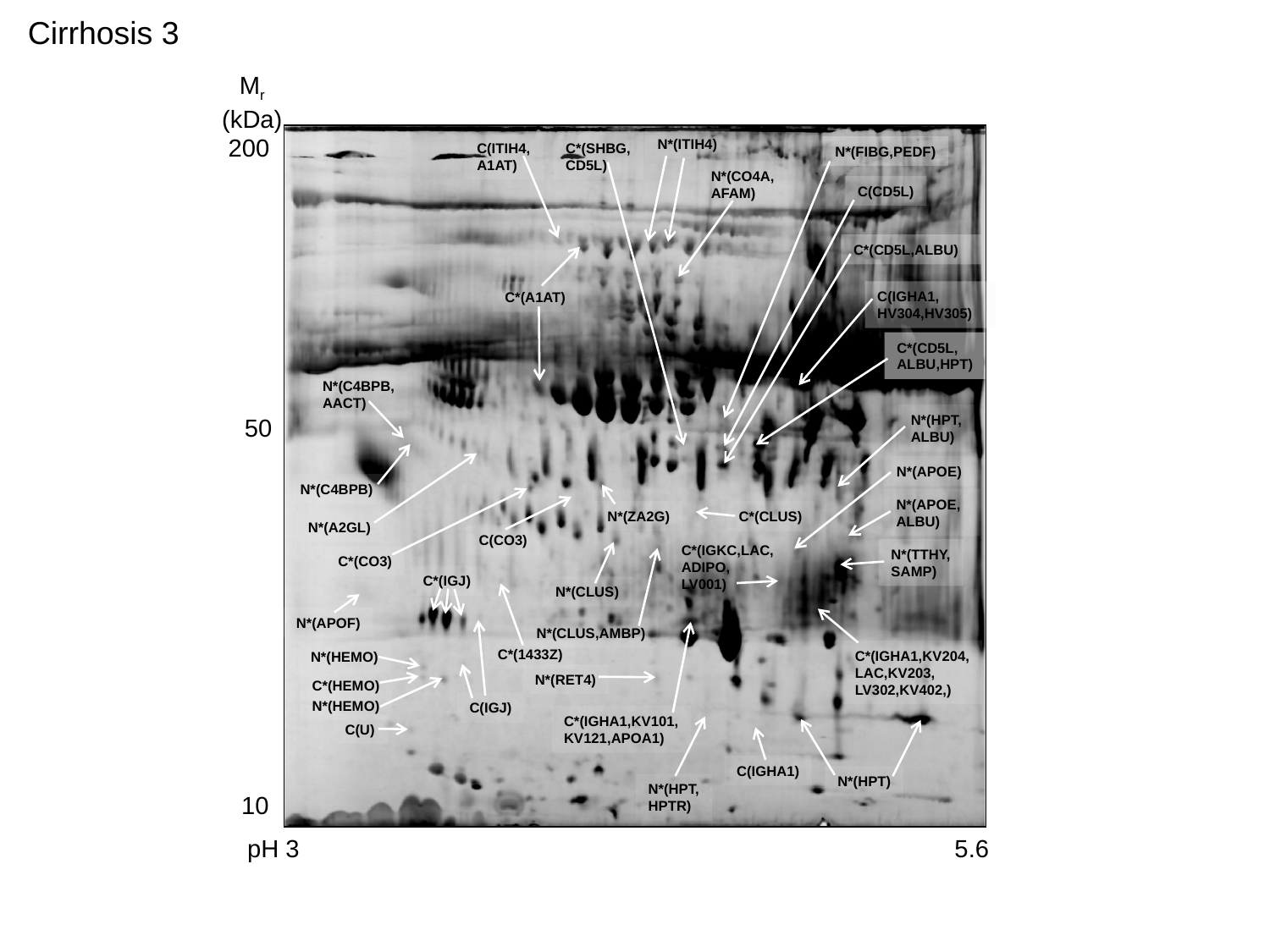

Cirrhosis 3
Mr
(kDa)
200
N*(ITIH4)
C(ITIH4,
A1AT)
C*(SHBG,
CD5L)
N*(FIBG,PEDF)
N*(CO4A,
AFAM)
C(CD5L)
C*(CD5L,ALBU)
C(IGHA1,
HV304,HV305)
C*(A1AT)
C*(CD5L,
ALBU,HPT)
N*(C4BPB,
AACT)
N*(HPT,
ALBU)
50
N*(APOE)
N*(C4BPB)
N*(APOE,
ALBU)
N*(ZA2G)
C*(CLUS)
N*(A2GL)
C(CO3)
C*(IGKC,LAC,
ADIPO,
LV001)
N*(TTHY,
SAMP)
C*(CO3)
C*(IGJ)
N*(CLUS)
N*(APOF)
N*(CLUS,AMBP)
C*(1433Z)
C*(IGHA1,KV204,
LAC,KV203,
LV302,KV402,)
N*(HEMO)
N*(RET4)
C*(HEMO)
N*(HEMO)
C(IGJ)
C*(IGHA1,KV101,
KV121,APOA1)
C(U)
C(IGHA1)
N*(HPT)
N*(HPT,
HPTR)
10
pH 3 5.6

## Slide 10
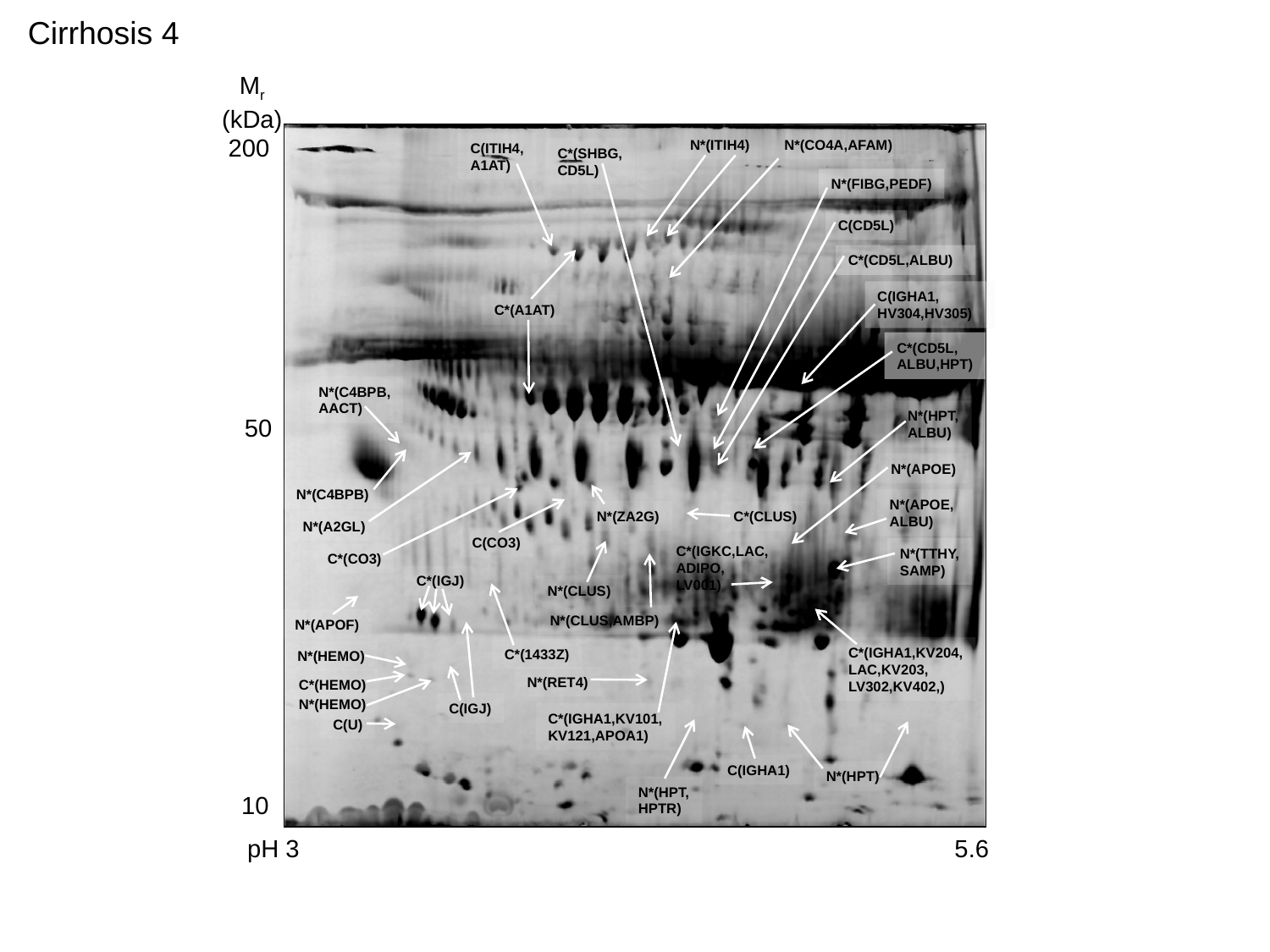

Cirrhosis 4
Mr
(kDa)
200
N*(CO4A,AFAM)
N*(ITIH4)
C(ITIH4,
A1AT)
C*(SHBG,
CD5L)
N*(FIBG,PEDF)
C(CD5L)
C*(CD5L,ALBU)
C(IGHA1,
HV304,HV305)
C*(A1AT)
C*(CD5L,
ALBU,HPT)
N*(C4BPB,
AACT)
N*(HPT,
ALBU)
50
N*(APOE)
N*(C4BPB)
N*(APOE,
ALBU)
N*(ZA2G)
C*(CLUS)
N*(A2GL)
C(CO3)
C*(IGKC,LAC,
ADIPO,
LV001)
N*(TTHY,
SAMP)
C*(CO3)
C*(IGJ)
N*(CLUS)
N*(CLUS,AMBP)
N*(APOF)
C*(IGHA1,KV204,
LAC,KV203,
LV302,KV402,)
C*(1433Z)
N*(HEMO)
N*(RET4)
C*(HEMO)
N*(HEMO)
C(IGJ)
C*(IGHA1,KV101,
KV121,APOA1)
C(U)
C(IGHA1)
N*(HPT)
N*(HPT,
HPTR)
10
pH 3 5.6

## Slide 11
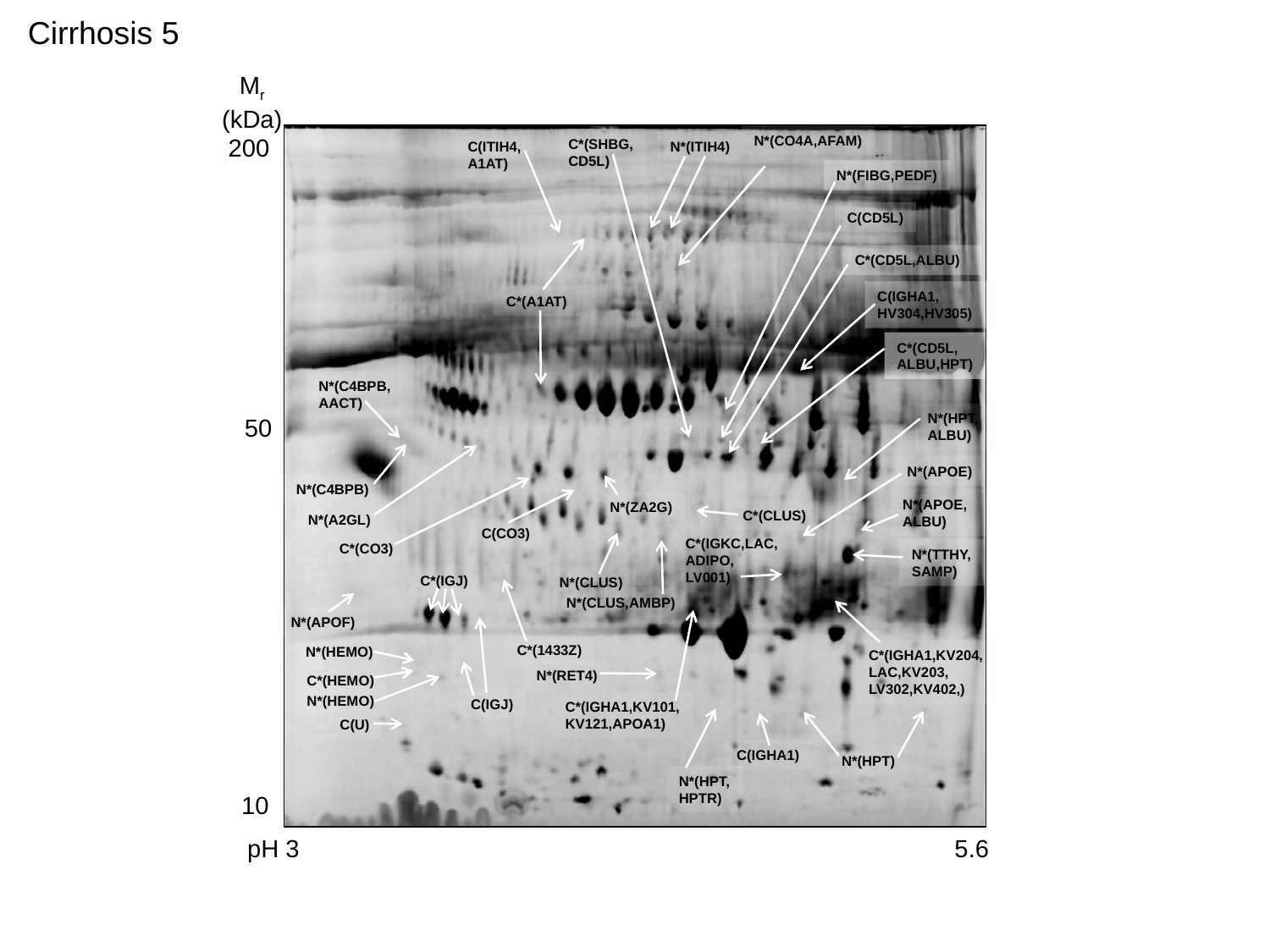

Cirrhosis 5
Mr
(kDa)
N*(CO4A,AFAM)
200
C*(SHBG,
CD5L)
N*(ITIH4)
C(ITIH4,
A1AT)
N*(FIBG,PEDF)
C(CD5L)
C*(CD5L,ALBU)
C(IGHA1,
HV304,HV305)
C*(A1AT)
C*(CD5L,
ALBU,HPT)
N*(C4BPB,
AACT)
N*(HPT,
ALBU)
50
N*(APOE)
N*(C4BPB)
N*(APOE,
ALBU)
N*(ZA2G)
C*(CLUS)
N*(A2GL)
C(CO3)
C*(IGKC,LAC,
ADIPO,
LV001)
C*(CO3)
N*(TTHY,
SAMP)
C*(IGJ)
N*(CLUS)
N*(CLUS,AMBP)
N*(APOF)
C*(1433Z)
N*(HEMO)
C*(IGHA1,KV204,
LAC,KV203,
LV302,KV402,)
N*(RET4)
C*(HEMO)
N*(HEMO)
C(IGJ)
C*(IGHA1,KV101,
KV121,APOA1)
C(U)
C(IGHA1)
N*(HPT)
N*(HPT,
HPTR)
10
pH 3 5.6
