# Supplementary material for: Discovery of Novel Biomarker Candidates for Liver Fibrosis in Hepatitis C Patients: A Preliminary Study
Source: PLoS One. 2012 Jun 26;7(6):e39603. doi: 10.1371/journal.pone.0039603 (PMC3383672; doi:10.1371/journal.pone.0039603)

Figure S2

MSMS spectra for proteins identified by a single peptide

All spectra were derived from Mascot. Peptide fragmentation patterns were generated using the observed singly charged *y*- or *b*-ions. Fragment ions minus H_2O and NH_3 were omitted.

Adiponectin (pI 5.39; 28.2 kDa)

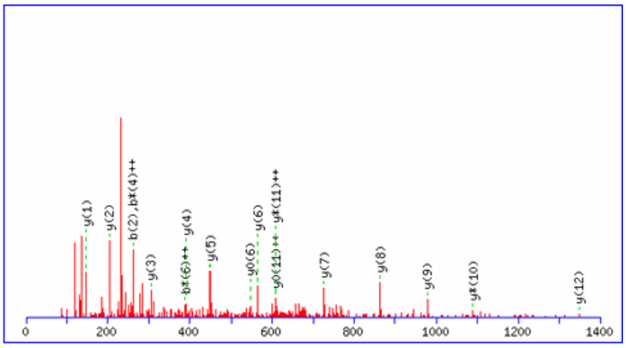

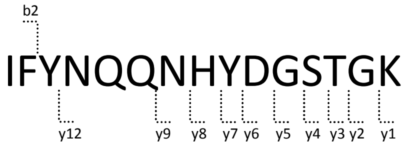

Afamin (pI 5.11; 80.0 kDa)

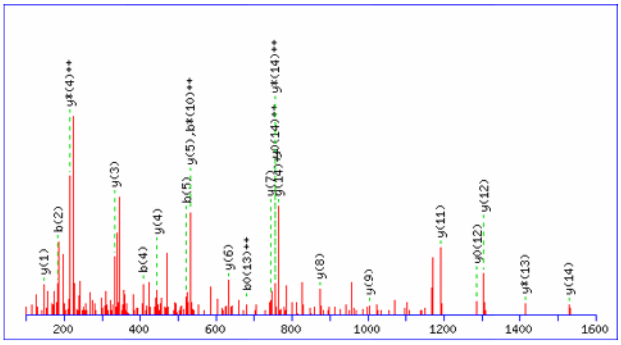

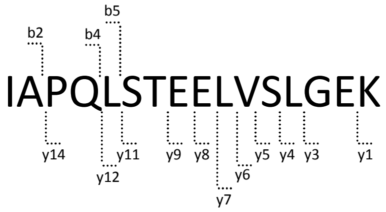

Albumin (pI 5.47; 39.7 kDa)

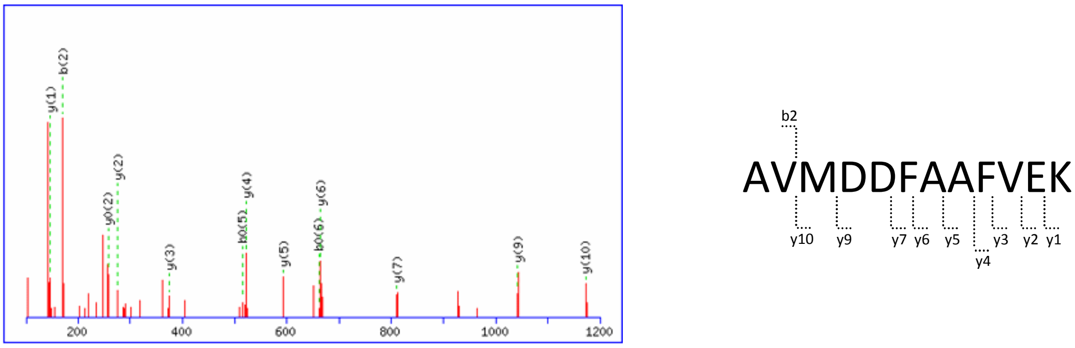

Albumin (pI 5.37; 45.9 kDa)

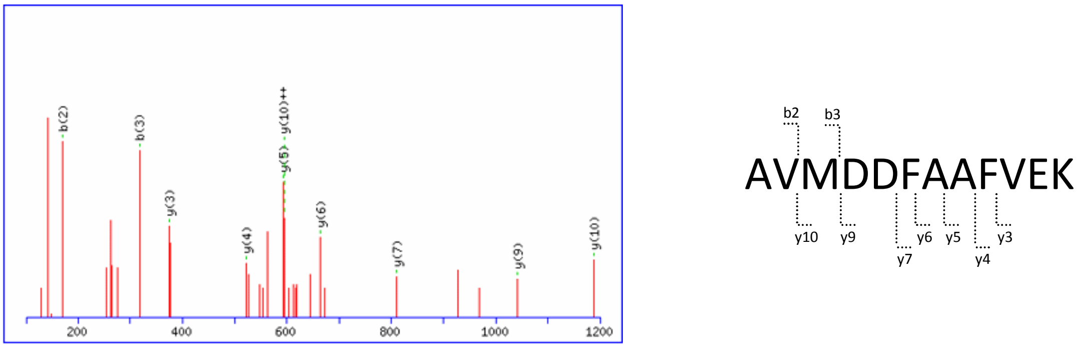

Alpha-1-antichymotrypsin (pI 4.66; 60.0 kDa)

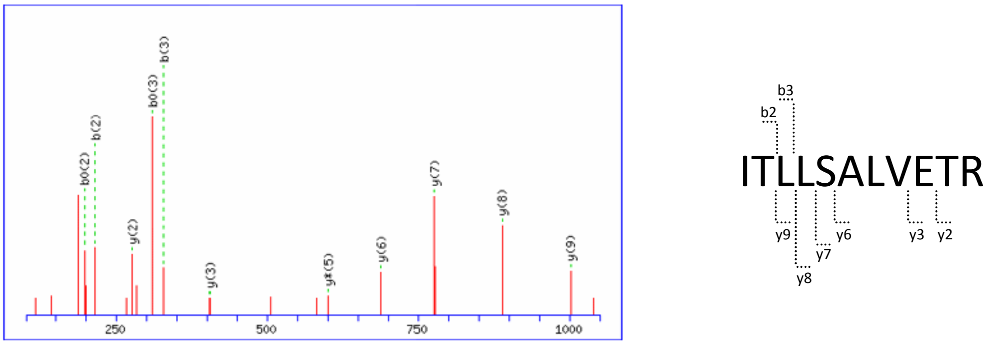

Apolipoprotein J (pI 5.14; 34.6 kDa)

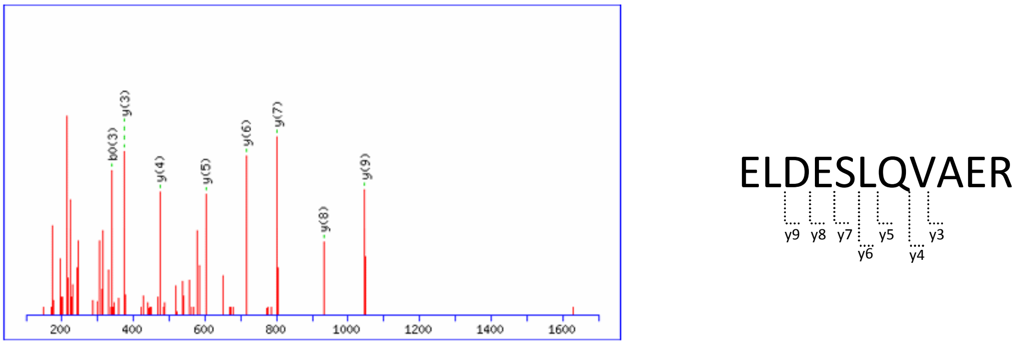

CD5 antigen-like (pI 5.31; 44.2 kDa)

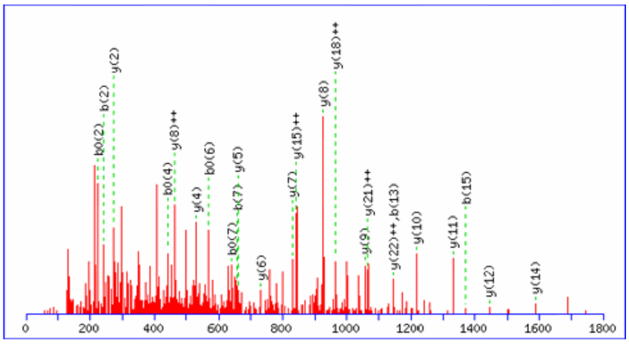

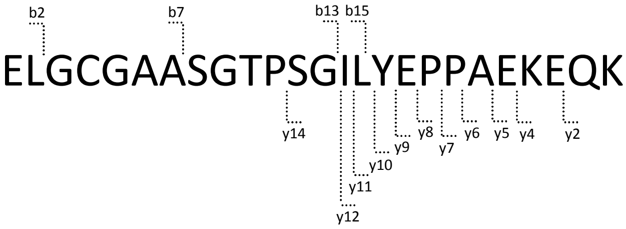

Corticosteroid-binding globulin (pI 4.69; 55.1 kDa)

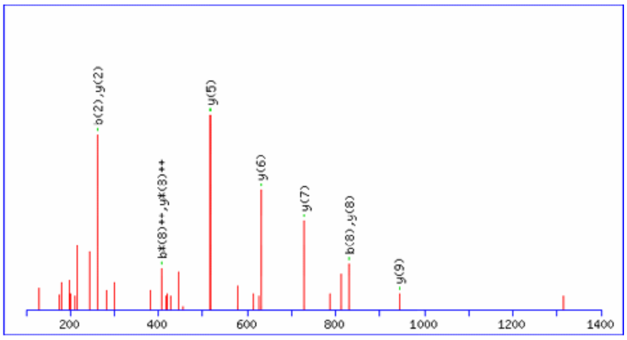

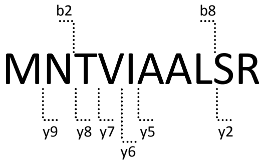

Haptoglobin (pI 5.37; 45.9 kDa)

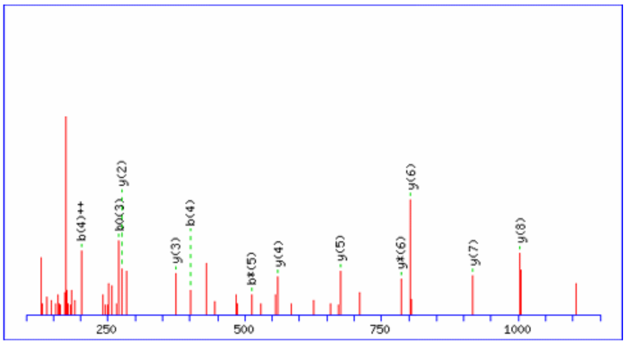

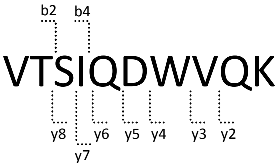

Haptoglobin-related protein (pI 5.21; 17.1 kDa)

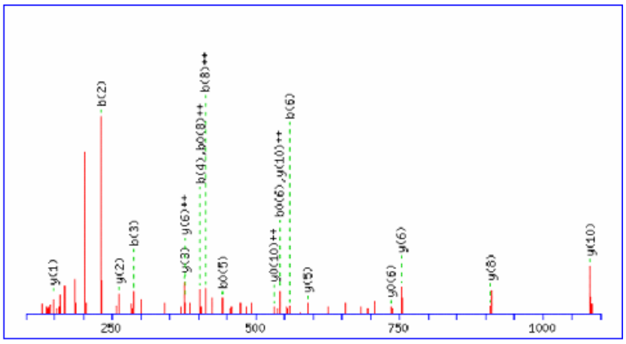

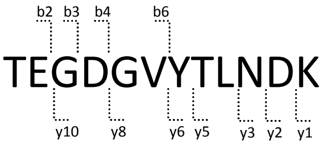

Hemopexin (pI 4.48; 20.2 kDa)

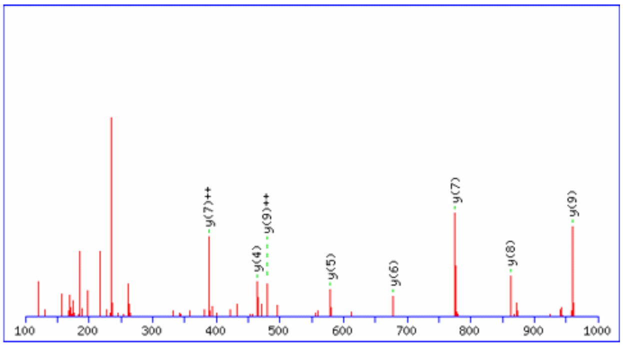

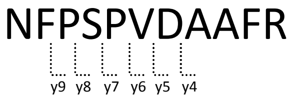

Hemopexin (pI 4.56; 19.3 kDa)

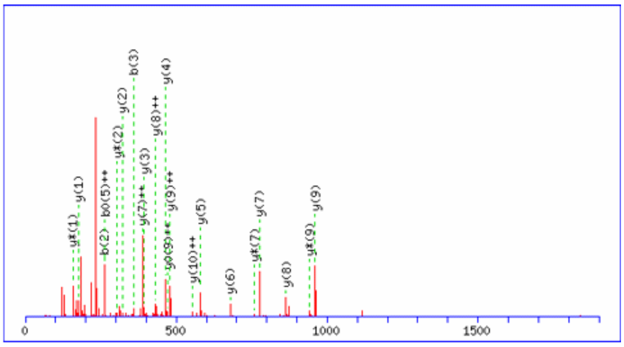

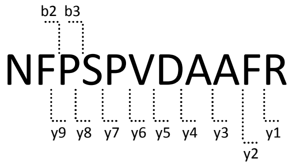

Hemopexin (pI 4.4; 18.9 kDa)

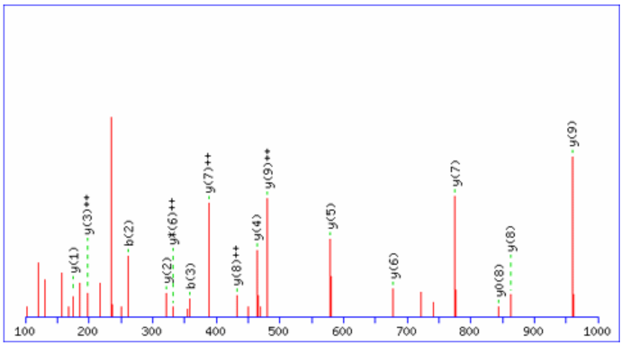

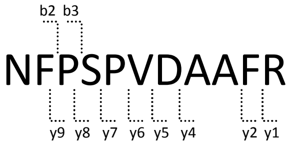

Ig alpha-1 chain C region (pI 5.35; 16.6 kDa)

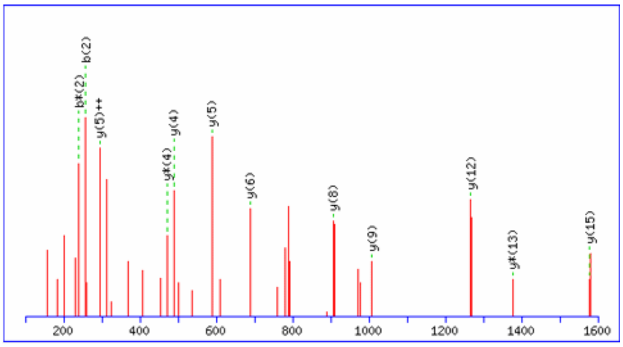

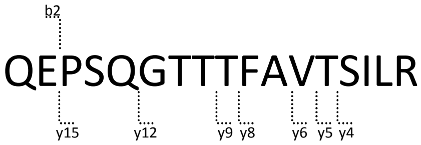

Ig heavy chain V-III region BRO (pI 5.42; 65.1 kDa)

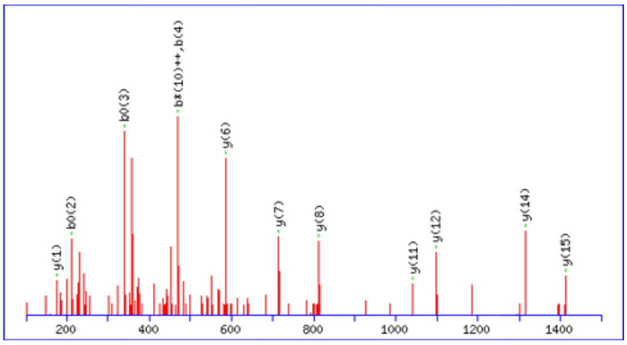

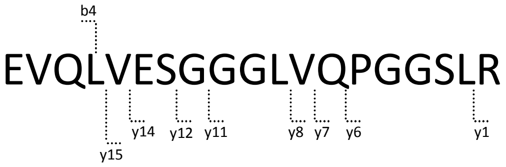

Ig heavy chain V-III region TIL (pI 5.42; 65.1 kDa)

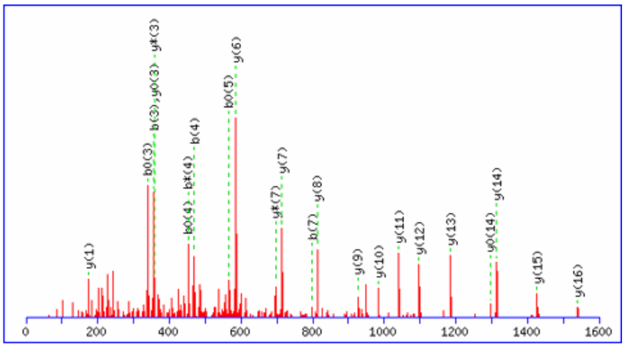

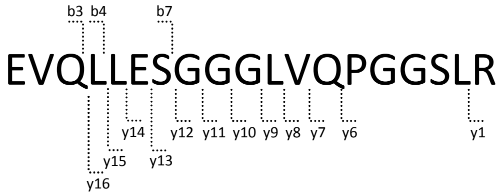

Ig lambda chain V-III region LOI (pI 5.44; 24.3 kDa)

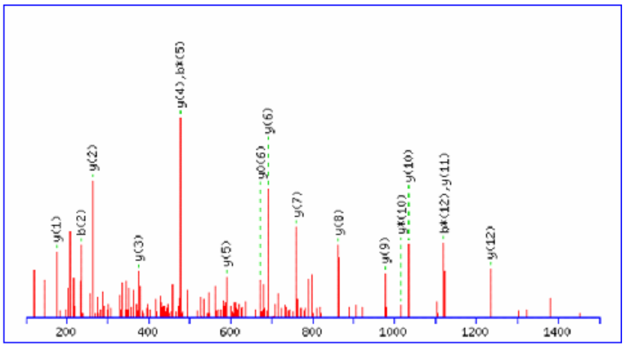

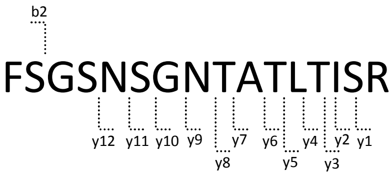

Ig Lambda chain V region 4a (pI 5.39; 28.2 kDa)

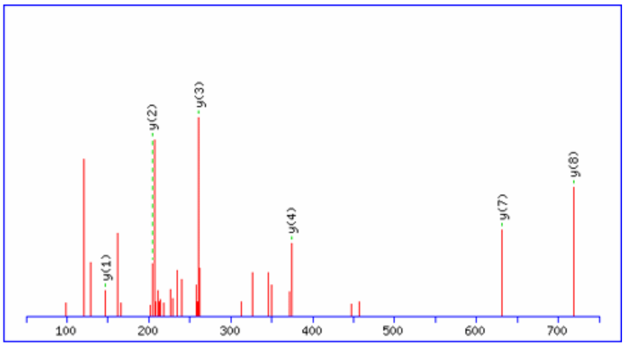

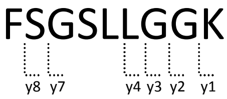

Immunoglobulin J chain (pI 4.7; 23.7 kDa)

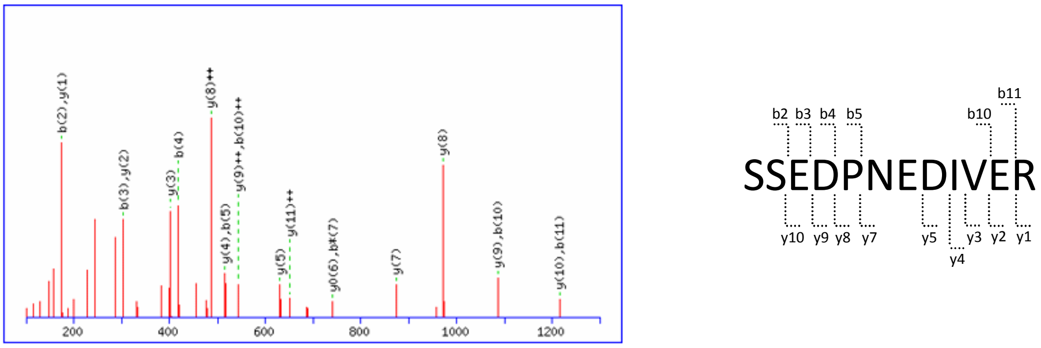

Immunoglobulin J chain (pI 4.67; 19.9 kDa)

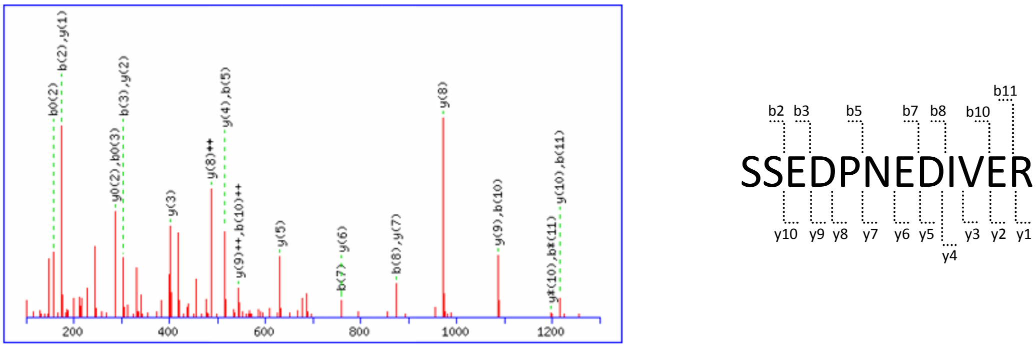

Lipid transfer inhibitor protein (pI 4.3; 25.8 kDa)

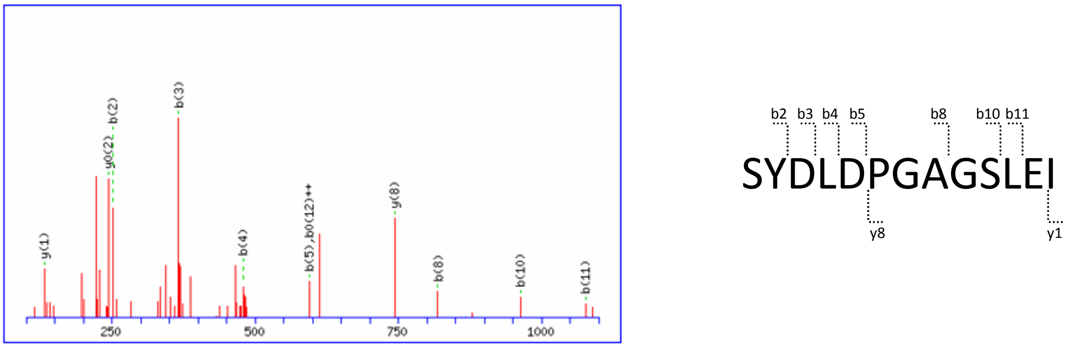

Sex hormone-binding globulin (pI 5.11; 45.0 kDa)

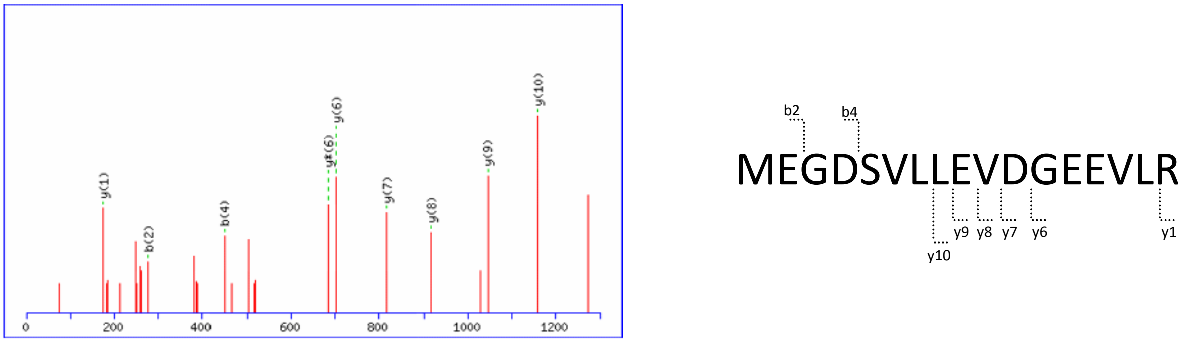

Supplement: Figure S2 — MSMS spectra for proteins identified by a single peptide. All spectra were derived from Mascot. Peptide fragmentation patterns were generated using the observed singly charged y- or b-ions. Fragment ions minus H2O and NH3 were omitted. (PDF) [file pone.0039603.s002.pdf]
