# Supplementary material for: Discovery of Novel Biomarker Candidates for Liver Fibrosis in Hepatitis C Patients: A Preliminary Study
Source: PLoS One. 2012 Jun 26;7(6):e39603. doi: 10.1371/journal.pone.0039603 (PMC3383672; doi:10.1371/journal.pone.0039603)
Supplement: Figure S3 — Haptoglobin expression analysis by 2-DE and Western blotting. (A) A decrease in haptoglobin in cirrhosis was observed in gel features at approximately 17 kDa and 40 kDa. The full length sequence of haptoglobin is shown with the alpha chain underlined and beta chain in bold. Peptides identified in the features at 17 kDa are highlighted in light grey and for the feature at 40 kDa in dark grey. (B) Magnified regions of the 2D gels at approximately 40 kDa showing the array of beta haptoglobin spots (arrowed). (C) Magnified regions of the 2D gels at approximately 17 kDa showing the alpha haptoglobin spots (circled). (D)Western blot of haptoglobin using plasma from 5 healthy individuals and 10 cirrhotic patients. This confirms the data in figures C and D that there is no significant difference in the expression of both alpha and beta haptoglobin when comparing plasma from normal individuals with plasma from cirrhotic patients. (E) Upper panel = Magnified regions of the 2D gels at approximately 40 kDa showing an isoform of beta haptoglobin (circled) between pH 5.46–5.49 to the right of the beta haptoglobin array. Lower panel = Zoomed in image of the beta haptoglobin feature between pH 5.46–5.49 (circled). (DOC) [file pone.0039603.s003.doc]

**Figure S3. Haptoglobin expression analysis by 2-DE and Western blotting.**

**A** A decrease in haptoglobin in cirrhosis was observed in gel features at approximately 17 kDa and 40 kDa. The full length sequence of haptoglobin is shown with the alpha chain underlined and beta chain in bold. Peptides identified in the features at 17 kDa are highlighted in light grey and for the feature at 40 kDa in dark grey

**B** Magnified regions of the 2D gels at approximately 40 kDa showing the array of beta haptoglobin spots (arrowed).

**C** Magnified regions of the 2D gels at approximately 17 kDa showing the alpha haptoglobin spots (circled).

**D** Western blot of haptoglobin using plasma from 5 healthy individuals and 10 cirrhotic patients. This confirms the data in figures C and D that there is no significant difference in the expression of both alpha and beta haptoglobin when comparing plasma from normal individuals with plasma from cirrhotic patients

**E** *Upper panel =* Magnified regions of the 2D gels at approximately 40 kDa showing an isoform of beta haptoglobin (circled) between pH 5.46 – 5.49 to the right of the beta haptoglobin array

*Lower panel =* Zoomed in image of the beta haptoglobin feature between pH 5.46 – 5.49 (circled)
