# Supplementary material for: Discovery of Novel Biomarker Candidates for Liver Fibrosis in Hepatitis C Patients: A Preliminary Study
Source: PLoS One. 2012 Jun 26;7(6):e39603. doi: 10.1371/journal.pone.0039603 (PMC3383672; doi:10.1371/journal.pone.0039603)
Supplement: Figure S4 — Western blot validation. Four plasma samples from controls (Ishak score 0) and two plasma samples from patients in each of the six Ishak stages of hepatic scarring (stages 1–6) were run on 17-well SDS-PAGE gels. Separated plasma proteins were electroblotted onto nitrocellulose membranes and probed with the following primary antibodies: afamin, adiponectin, IgJ, hemopexin, 14-3-3zeta, apolipoprotein E (Apo E), apolipoprotein C3 (Apo C3), beta 2 glycoprotein-I (B2GPI), inter-alpha-trypsin inhibitor heavy chain H4 (ITIH4), CD5L and zinc-alpha-2-glycoprotein (ZAG). Bands were detected with ECL Plus. (DOC) [file pone.0039603.s004.doc]

**Figure S4. Western blot validation**

Four plasma samples from controls (Ishak score 0) and two plasma samples from patients in each of the six Ishak stages of hepatic scarring (stages 1-6) were run on 17-well SDS-PAGE gels. Separated plasma proteins were electroblotted onto nitrocellulose membranes and probed with the following primary antibodies: afamin, adiponectin, IgJ, hemopexin, 14-3-3zeta, apolipoprotein E (Apo E), apolipoprotein C3 (Apo C3), beta 2 glycoprotein-I (B2GPI), inter-alpha-trypsin inhibitor heavy chain H4 (ITIH4), CD5L and zinc-alpha-2-glycoprotein (ZAG). Bands were detected with ECL Plus.
