# Supplementary material for: Discovery of Novel Biomarker Candidates for Liver Fibrosis in Hepatitis C Patients: A Preliminary Study
Source: PLoS One. 2012 Jun 26;7(6):e39603. doi: 10.1371/journal.pone.0039603 (PMC3383672; doi:10.1371/journal.pone.0039603)

**Figure S5. Ingenuity Pathway Analysis**

Differentially expressed proteins were analysed using the Ingenuity Pathway Analysis software. Potential protein interactions are shown. Identified proteins are coloured and labelled with their gene names as shown in Table S2. Potential interacting partners which were not identified in the 2-DE study are shown in white.

Solid lines (green, red, white, pink) represent direct interactions, dashed lines (yellow, grey) represent indirect interactions. Arrows (white, yellow, red, pink) from one protein node to another indicates that the node acts on the other node. Lines without arrowheads (green) represent binding. Lines with a small perpendicular line at the end (grey) represent inhibition. Proteins identified by differential analysis are shown as coloured nodes whereas unidentified proteins are white.


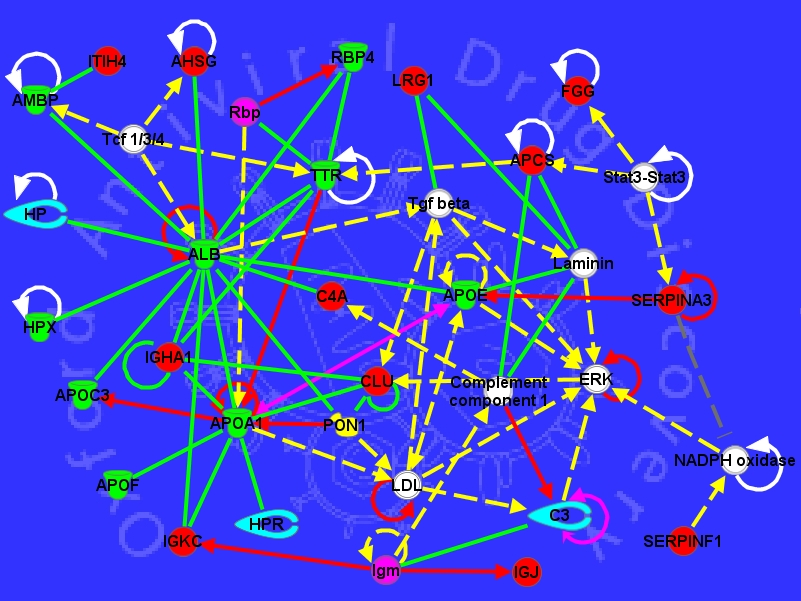

Supplement: Figure S5 — Ingenuity Pathway Analysis. Differentially expressed proteins were analysed using the Ingenuity Pathway Analysis software. Potential protein interactions are shown. Identified proteins are coloured and labelled with their gene names as shown in Table S1. Potential interacting partners which were not identified in the 2-DE study are shown in white. Solid lines (green, red, white, pink) represent direct interactions, dashed lines (yellow, grey) represent indirect interactions. Arrows (white, yellow, red, pink) from one protein node to another indicates that the node acts on the other node. Lines without arrowheads (green) represent binding. Lines with a small perpendicular line at the end (grey) represent inhibition. Proteins identified by differential analysis are shown as coloured nodes whereas unidentified proteins are white. (DOC) [file pone.0039603.s005.doc]
