# Supplementary material for: Discovery of Novel Biomarker Candidates for Liver Fibrosis in Hepatitis C Patients: A Preliminary Study
Source: PLoS One. 2012 Jun 26;7(6):e39603. doi: 10.1371/journal.pone.0039603 (PMC3383672; doi:10.1371/journal.pone.0039603)
Supplement: Method S1 — Biomarker validation. (DOC) [file pone.0039603.s006.doc]

**Method S1**

**Biomarker validation.** Mouse anti adiponectin was purchased from Millipore (Watford, UK). Rabbit anti procollagen type III and mouse anti afamin were purchased from Abnova (Taipei, Taiwan). Goat anti beta 2 glycoprotein-I was purchased from VH Bio (Gateshead, UK). Rat anti apolipoprotein L1 was kindly provided by Prof. Etienne Pays (Universite Libre de Bruxelles, Belgium). Rabbit anti alpha 2 macroglobulin, rabbit anti haptoglobin and rabbit anti apolipoprotein A1 were purchased from Dako (Ely, UK). Goat anti apolipoprotein C-III, rabbit anti apolipoprotein E and rabbit anti CD5L were purchased from AbD Serotec (Oxford, UK). Goat anti lipid transfer inhibitor protein, mouse anti complement C3d, goat anti inter-alpha-trypsin inhibitor heavy chain H4, mouse anti apolipoprotein J, goat anti hemopexin, rabbit anti TIMP1, goat anti corticosteroid-binding globulin, mouse anti zinc-alpha-2-glycoprotein, goat anti IgJ, rabbit anti 14-3-3zeta and HRP-conjugated anti goat antibodies were from Santa Cruz Biotechnology (Santa Cruz, CA). HRP-conjugated anti-mouse and anti-rabbit antibodies were from GE Healthcare (Bucks, UK). HRP-conjugated anti-rat antibody was from Abcam (Cambridge, UK). Hyaluronic acid was measured using a competitive ELISA according to the manufacturer’s recommendation (Echelon, Salt Lake City, UT). Hyaluronic acid levels were determined in the plasma samples of 24 patients and the number of patients used for each of the 7 Ishak stages is shown in parentheses: 0[4]; 1[3]; 2[3], 3[4], 4[2], 5[4], 6[4]. The following were measured in the John Radcliffe Hospital, Oxford and are shown in Table S1: Alanine aminotransferase (IU/L), Albumin (g/L), Alkaline phosphatase (IU/L), Bilirubin (μmol/L), Creatinine (mmol/L), Gamma glutamyl transpeptidase (IU/L), HCV genotype, INR ratio, Platelet count, Prothrombin time PT (seconds), Viral load by PCR (IU/ml), Number of tracts (average 12.7), Size of the core (mm), Portal inflammation (out of 4), Interface hepatitis (out of 4), Confluent necrosis (out of 6), Lobular inflammation (out of 4), Total Ishak grade (out of 18), MELD score for cirrhotic patients and Child Pugh for cirrhotic patients. Total bilirubin levels were determined in the plasma samples of 36 patients and the number of patients used for each of the 7 Ishak stages is shown in parentheses: 0[2]; 1[5]; 2[9], 3[6], 4[2], 5[5], 6[7]. Gamma glutamyltranspeptidase levels were determined in the plasma samples of 33 patients and the number of patients used for each of the 7 Ishak stages is shown in parentheses: 0[2]; 1[4]; 2[8], 3[5], 4[2], 5[4], 6[8].
