# Supplementary material for: Discovery of Novel Biomarker Candidates for Liver Fibrosis in Hepatitis C Patients: A Preliminary Study
Source: PLoS One. 2012 Jun 26;7(6):e39603. doi: 10.1371/journal.pone.0039603 (PMC3383672; doi:10.1371/journal.pone.0039603)
Supplement: Table S2 — Differentially expressed proteins identified in plasma samples of healthy controls versus cirrhotic patients. Entries in blue indicate proteins which had the highest score within a protein spot. Among these, the protein names and entries in bold are novel and were not seen in our earlier study. [13] AN, Swiss-Prot accession number; N, feature present in plasma from healthy controls; C, feature present in plasma from cirrhosis patients. Fold change refers to proteins that were differentially expressed by 2-fold or more when comparing plasma gels from healthy controls with cirrhosis. The numerical values shown in parentheses for fold change indicate features that were present in both controls and cirrhosis but expressed to a higher extent in the indicated stage. For cases where no numerical value is shown for fold change, the feature was only present in the indicated stage. pI, isoelectric point on gel as determined by the image analysis software using calibrated landmarks; MWt, molecular weight on gel as determined by the image analysis software using calibrated landmarks. The number of MS/MS peptide matches, percentage sequence coverage and protein score were determined by the Mascot Daemon search engine. Protein functions have been adapted from the ExPASy website. (DOC) [file pone.0039603.s008.doc]

**Table S2**

Differentially expressed proteins identified in plasma samples of healthy controls versus cirrhotic patients.

Entries in blue indicate proteins which had the highest score within a protein spot. Among these, the protein names and entries in bold are novel and were not seen in our earlier study.^13^

AN, Swiss-Prot accession number

N, feature present in plasma from healthy controls

C, feature present in plasma from cirrhosis patients

Fold change refers to proteins that were differentially expressed by 2-fold or more when comparing plasma gels from healthy controls with cirrhosis. The numerical values shown in parentheses for fold change indicate features that were present in both controls and cirrhosis but expressed to a higher extent in the indicated stage. For cases where no numerical value is shown for fold change, the feature was only present in the indicated stage

pI, isoelectric point on gel as determined by the image analysis software using calibrated landmarks

MWt, molecular weight on gel as determined by the image analysis software using calibrated landmarks

The number of MS/MS peptide matches, percentage sequence coverage and protein score were determined by the Mascot Daemon search engine.

Protein functions have been adapted from the ExPASy website

| **Protein Name** | **AN** | **Gene name** | **Swiss-Prot entry name** | **Fold change** | **pI** | **MWt (kDa)** | **No of peptides** | **Sequence coverage (%)** | **Protein Score** | **Function** |
| --- | --- | --- | --- | --- | --- | --- | --- | --- | --- | --- |
| **14-3-3 protein zeta/delta** | P63104 | YWHAZ | 1433Z | **C* (2.89)** | **4.72** | **25.8** | **3** | **15** | **87** | Adapter protein implicated in signalling pathway regulation. Binds proteins usually by recognition of phospho-serine/threonine which can modulate binding partner activity |
| **Adiponectin** | Q15848 | ADIPOQ | ADIPO | **C* (2.93)** | **5.39** | **28.2** | **1** | **6** | **46** | Adipokine involved in fat metabolism control & insulin sensitivity with anti-diabetic, anti-atherogenic & anti-inflammatory activities. Activates in liver, enhancing glucose utilization & fatty-acid combustion. Antagonizes TNF-alpha in tissues such as liver. Inhibits endothelial NFkB signaling. May play role in cell growth, angiogenesis & tissue remodeling by binding & sequestering growth factors |
| **Afamin** | P43652 | AFM | AFAM | **N* (2.06)** | **5.11** | **80.0** | **1** | **2** | **41** | Possible role in the transport of yet unknown ligand |
| Albumin | P02768 | ALB | ALBU | N* (3.86) | 5.47 | 39.7 | 1 | 1 | 37 | Main plasma protein. Regulates blood colloidal osmotic pressure |
|  |  |  |  | N* (2.50) | 5.5 | 32.6 | 3 | 3 | 119 |  |
|  |  |  |  | C* (2.75) | 5.31 | 41.4 | 3 | 3 | 99 |  |
|  |  |  |  | C* (3.23) | 5.37 | 45.9 | 1 | 1 | 56 |  |
| Alpha-1-antichymotrypsin | P01011 | SERPINA3 | AACT | N | 4.63 | 62.5 | 3 | 9 | 158 | Can inhibit neutrophil cathepsin G & mast cell chymase, both of which can convert angiotensin-1 to angiotensin-2 |
|  |  |  |  | N | 4.66 | 60.0 | 1 | 2 | 40 |  |
|  |  |  |  | N* (2.15) | 4.46 | 45.9 | 2 | 6 | 68 |  |
| **Alpha-1-antitrypsin** | P01009 | SERPINA1 | A1AT | **N** | **4.89** | **49.2** | **2** | **4** | **69** | Serine protease inhibitor. Primary target is elastase with moderate affinity for plasmin and thrombin. Irreversibly inhibits trypsin, chymotrypsin and plasminogen activator. Aberrant form decreases coagulation time and has proteolytic activity against plasmin |
|  |  |  |  | **C** | **4.85** | **108.6** | **3** | **6** | **90** |  |
|  |  |  |  | **C* (2.00)** | **4.9** | **105.7** | **16 (15)** | **35** | **332** |  |
|  |  |  |  | C* (2.42) | 4.8 | 60.1 | 17 (15) | 35 | 568 |  |
| **Alpha-2-HS-glycoprotein** | P02765 | AHSG | FETUA | N | 4.63 | 62.5 | 5 | 20 | 85 | Promotes endocytosis, possesses opsonic properties & influences mineral phase of bone. Shows affinity for calcium & barium ions |
|  |  |  |  | N | 4.66 | 60.0 | 2 | 8 | 43 |  |
|  |  |  |  | **N** | **4.69** | **55.1** | **4** | **17** | **91** |  |
| AMBP | P02760 | AMBP | AMBP | N* (2.26) | 5.06 | 30.6 | 2 | 8 | 68 | Inter-alpha-trypsin inhibitor inhibits trypsin, plasmin, and lysosomal granulocytic elastase. Inhibits calcium oxalate crystallization |
| Apolipoprotein A-I | P02647 | APOA1 | APOA1 | C* (2.98) | 5.13 | 23.7 | 2 | 9 | 121 | Role in cholesterol reverse transport from tissues to liver for excretion. Promotes cholesterol efflux from tissues & acts as a cofactor for lecithin cholesterol acyltransferase |
| **Apolipoprotein C-III** | P02656 | APOC3 | APOC3 | **N** | **4.61** | **15.5** | **2** | **19** | **151** | Inhibits lipoprotein lipase & hepatic lipase. Decreases uptake of lymph chylomicrons by hepatic cells. Delays catabolism of triglyceride-rich particles |
|  |  |  |  | **N** | **4.62** | **15.1** | **2** | **19** | **117** |  |
| **Apolipoprotein E** | P02649 | APOE | APOE | **N* (2.01)** | **5.42** | **30.3** | **12** | **43** | **373** | Mediates binding, internalization, catabolism of lipoprotein particles. Can serve as ligand for LDL (apo B/E) receptor & hepatic apo-E receptor (chylomicron remnant) |
|  |  |  |  | **N* (2.50)** | **5.5** | **32.6** | **10** | **33** | **271** |  |
| **Apolipoprotein J** | P10909 | CLU | CLUS | **N* (2.52)** | **4.96** | **31.4** | **4 (3)** | **5** | **125** | Binds to cells, membranes and hydrophobic proteins. Associated with apoptosis |
|  |  |  |  | **N* (2.26)** | **5.06** | **30.6** | **3** | **5** | **108** |  |
|  |  |  |  | **N** | **4.9** | **34.7** | **2** | **6** | **67** |  |
|  |  |  |  | C* (2.61) | 5.14 | 34.6 | 1 | 2 | 58 |  |
| **C4b-binding protein beta chain** | P20851 | C4BPB | C4BPB | **N* (2.15)** | **4.46** | **45.9** | **4** | **19** | **76** | Controls classical pathway of complement activation. Binds as cofactor to C3b/C4b inactivator (C3bINA), then hydrolyzes complement fragment C4b. Accelerates degradation of C4bC2a complex (C3 convertase) by dissociating complement fragment C2a. Interacts with anticoagulant protein S & serum amyloid P |
|  |  |  |  | **N* (2.13)** | **4.48** | **45.3** | **3** | **13** | **80** |  |
| CD5 antigen-like | O43866 | CD5L | CD5L | C* (4.40) | 5.11 | 45.0 | 1 | 5 | 33 | May play role in immune system regulation. May play role as apoptosis inhibitor |
|  |  |  |  | C | 5.31 | 44.2 | 1 | 7 | 48 |  |
|  |  |  |  | C* (2.75) | 5.31 | 41.4 | 9 | 39 | 201 |  |
|  |  |  |  | C* (3.23) | 5.37 | 45.9 | 6 | 20 | 180 |  |
| **Complement C3 (C3dg)** | P01024 | C3 | CO3 | C* (2.23) | 4.79 | 39.2 | 8 | 5 | 186 | Role in complement system activation. Processing by C3 convertase is central reaction |
|  |  |  |  | **C** | **4.88** | **37.7** | **4** | **3** | **146** |  |
| Complement C4-A | P0C0L4 | C4A | CO4A | N* (2.06) | 5.11 | 80.0 | 12 (11) | 9 | 399 | Role in complement classical pathway activation. Derived from proteolytic degradation of complement C4, C4a anaphylatoxin is a mediator of local inflammatory process |
| **Corticosteroid-binding globulin** | P08185 | SERPINA6 | CBG | **N** | **4.66** | **60.0** | **4** | **8** | **133** | Major blood transport protein for glucocorticoids & progestins |
|  |  |  |  | N | 4.69 | 55.1 | 1 | 2 | 54 |  |
| **Fibrinogen gamma chain** | P02679 | FGG | FIBG | **N* (2.06)** | **5.31** | **50.7** | **4** | **13** | **88** | Yields monomers that polymerize into fibrin & acts as cofactor in platelet aggregation |
| **Haptoglobin** | P00738 | HP | HPT | **N* (3.86)** | **5.47** | **39.7** | **12 (11)** | **24** | **339** | Combines with free plasma hemoglobin, preventing loss of iron through kidneys and protecting kidneys from damage by hemoglobin |
|  |  |  |  | N* (3.18) | 5.68 | 17.1 | 3 | 7 | 88 |  |
|  |  |  |  | N* (2.61) | 5.21 | 17.1 | 4 | 12 | 74 |  |
|  |  |  |  | N* (2.94) | 5.41 | 17.0 | 3 | 9 | 79 |  |
|  |  |  |  | N | 5.42 | 17.1 | 4 | 10 | 101 |  |
|  |  |  |  | C* (3.23) | 5.37 | 45.9 | 1 | 2 | 51 |  |
| **Haptoglobin-related protein** | P00739 | HPR | HPTR | **N* (2.61)** | **5.21** | **17.1** | **1** | **3** | **30** | Haptoglobin-related protein |
| **Hemopexin** | P02790 | HPX | HEMO | **N* (2.17)** | **4.48** | **20.2** | **1** | **2** | **49** | Binds heme & transports it to liver for breakdown & iron recovery, after which free hemopexin returns to circulation |
|  |  |  |  | **N*(2.01)** | **4.56** | **19.3** | **1** | **2** | **40** |  |
|  |  |  |  | **C* (2.58)** | **4.4** | **18.9** | **1** | **2** | **44** |  |
| Ig alpha-1 chain C region | P01876 | IGHA1 | IGHA1 | C | 5.42 | 65.1 | 13 (11) | 50 | 325 | Ig alpha is major immunoglobulin class in body secretions. Defends against local infection & prevents access of foreign antigens to general immunologic system |
|  |  |  |  | C | 5.35 | 16.6 | 1 | 4 | 41 |  |
|  |  |  |  | C* (2.25) | 5.44 | 24.3 | 7 (6) | 68 | 309 |  |
|  |  |  |  | C* (2.98) | 5.13 | 23.7 | 6 | 80 | 272 |  |
| Ig heavy chain V-III region BRO | P01766 |  | HV305 | C | 5.42 | 65.1 | 1 | 15 | 48 | V-III region BRO of Ig heavy chain |
| Ig heavy chain V-III region TIL | P01765 |  | HV304 | C | 5.42 | 65.1 | 1 | 16 | 70 | V-III region TIL of Ig heavy chain |
| Ig kappa chain C region | P01834 | IGKC | IGKC | N | 5.47 | 26.9 | 3 | 44 | 143 | Ig kappa chain C region |
|  |  |  |  | C* (2.93) | 5.39 | 28.2 | 2 | 30 | 78 |  |
| Ig kappa chain V-I region AG | P01593 |  | KV101 | C* (2.98) | 5.13 | 23.7 | 3 | 31 | 163 | V-I region AG of Ig kappa chain |
| Ig kappa chain V-I region Ni | P01613 |  | KV121 | C* (2.98) | 5.13 | 23.7 | 2 (1) | 30 | 140 | V-I region Ni of Ig kappa chain |
| Ig kappa chain V-II region MIL | P01616 |  | KV203 | C* (2.25) | 5.44 | 24.3 | 2 (1) | 33 | 96 | V-II region MIL of Ig kappa chain |
| Ig kappa chain V-II region TEW | P01617 |  | KV204 | C* (2.25) | 5.44 | 24.3 | 3 | 32 | 122 | V-II region TEW of Ig kappa chain |
| Ig lambda chain C regions | P01842 | IGLC1/2/3 | LAC | N | 5.47 | 26.9 | 2 | 28 | 118 | Ig lambda chain C regions |
|  |  |  |  | C* (2.93) | 5.39 | 28.2 | 2 | 28 | 74 |  |
|  |  |  |  | C* (2.25) | 5.44 | 24.3 | 3 | 46 | 91 |  |
| Ig lambda chain V region 4A | P04211 |  | LV001 | C* (2.93) | 5.39 | 28.2 | 1 | 7 | 34 |  |
| Ig lambda chain V-III region LOI | P80748 |  | LV302 | C* (2.25) | 5.44 | 24.3 | 1 | 14 | 87 | V-III region LOI of Ig lamda chain |
| **Immunoglobulin J chain** | P01591 | IGJ | IGJ | **C* (2.42)** | **4.47** | **24.2** | **5** | **43** | **146** | Links two monomer units of IgM or IgA. For IgM, J chain-joined dimer is a nucleating unit for IgM pentamer. For IgA, it induces larger polymers |
|  |  |  |  | **C* (2.67)** | **4.58** | **23.7** | **2** | **14** | **47** |  |
|  |  |  |  | **C** | **4.7** | **23.7** | **1** | **8** | **79** |  |
|  |  |  |  | **C* (6.21)** | **4.67** | **23.1** | **5** | **35** | **157** |  |
|  |  |  |  | **C** | **4.67** | **19.9** | **1** | **8** | **84** |  |
| Inter-alpha-trypsin inhibitor heavy chain H4 | Q14624 | ITIH4 | ITIH4 | N* (2.10) | 5.05 | 99.8 | 12 | 16 | 305 | May be involved in acute phase reactions |
|  |  |  |  | N* (2.29) | 5.09 | 99.8 | 16 | 15 | 469 |  |
|  |  |  |  | C | 4.85 | 108.6 | 4 | 5 | 111 |  |
| **Leucine-rich alpha-2-glycoprotein** | P02750 | LRG1 | A2GL | **N* (3.39)** | **4.68** | **45.1** | **5** | **16** | **197** | Involved in protein-protein interactions, signal transduction, cell adhesion & development |
| **Lipid transfer inhibitor protein** | Q13790 | APOF | APOF | **N* (2.01)** | **4.3** | **25.8** | **1** | **4** | **37** | Associates with LDL. Inhibits cholesteryl ester transfer protein activity & regulator of cholesterol transport |
| Paraoxonase/ arylesterase 1 | P27169 | PON1 | PON1 | N | 4.86 | 50.0 | 7 | 21 | 199 | May mediate enzymatic protection of low density lipoproteins against oxidative modification |
|  |  |  |  | N | 4.87 | 48.8 | 10 | 29 | 231 |  |
| Pigment epithelium-derived factor | P36955 | SERPINF1 | PEDF | N* (2.06) | 5.31 | 50.7 | 3 | 13 | 63 | Induces extensive neuronal differentiation in retinoblastoma cells. Inhibitor of angiogenesis. Exhibits no serine protease inhibitory activity |
| **Retinol-binding protein 4** | P02753 | RET4 | RET4 | **N* (2.54)** | **5.05** | **19.1** | **3** | **15** | **87** | Delivers retinol from liver to peripheral tissues. RBP-retinol interacts with transthyretin preventing its loss by filtration through kidneys |
| Serum amyloid P-component | P02743 | APCS | SAMP | N* (2.69) | 5.48 | 28.3 | 3 | 16 | 81 | Interact with DNA / histones and may scavenge nuclear material released from damaged cells. May act as calcium-dependent lectin |
| **Sex hormone-binding globulin** | P04278 | SHBG | SHBG | **C* (4.40)** | **5.11** | **45.0** | **1** | **4** | **42** | Androgen transport protein. May be involved in receptor mediated processes. Regulates clearance of steroid hormones |
| Transthyretin | P02766 | TTR | TTHY | N* (2.69) | 5.48 | 28.3 | 23 (9) | 69 | 599 | Thyroid hormone-binding protein. May transport thyroxine from bloodstream to brain |
|  |  |  |  | N | 5.47 | 26.9 | 7 (6) | 53 | 232 |  |
| Zinc-alpha-2-glycoprotein | P25311 | AZGP1 | ZA2G | N* (2.01) | 4.93 | 39.4 | 6 (5) | 18 | 142 | Stimulates lipid degradation in adipocytes and causes fat losses in some cancers. May bind polyunsaturated fatty acids |
| Unidentified | - | - | U | N | 4.92 | 13.4 | _ | _ | _ | - |
|  |  |  |  | N | 5.04 | 13.3 |  |  |  |  |
|  |  |  |  | C | 4.31 | 15.6 |  |  |  |  |
