# Supplementary material for: Discovery of Novel Biomarker Candidates for Liver Fibrosis in Hepatitis C Patients: A Preliminary Study
Source: PLoS One. 2012 Jun 26;7(6):e39603. doi: 10.1371/journal.pone.0039603 (PMC3383672; doi:10.1371/journal.pone.0039603)
Supplement: Table S3 — Potential involvement of a selection of the novel proteins in hepatic scarring. (DOC) [file pone.0039603.s009.doc]

**Table S3**

**Potential involvement of a selection of the novel proteins in hepatic scarring**

| **Novel fibrosis marker** | **Potential involvement** |
| --- | --- |
| Apolipoprotein C-III | Has never been suggested as a liver scarring biomarker but its levels have been shown to be lower in patients infected with HCV than those with HBV.^1^ Improvement of liver function and fibrosis in mice with hepatic scarring is associated with an increase in apolipoprotein C-III^2^ which is consistent with the higher levels of the apolipoprotein we observed in healthy individuals in our study. |
| Apolipoprotein E | Is important for HCV assembly and release of infectious viral particles.^3^ Although apolipoprotein E has never been described as a marker for hepatic scarring, its polymorphism may influence the progression of primary biliary cirrhosis.^4^ |
| Hemopexin | Has been shown to increase in serum from patients with hepatic scarring.^5^ Our 2-DE data supports this change but also shows for the first time additional gel features with hemopexin that are decreasing in cirrhosis suggesting potential post-translational modification. In our Western blot analysis we were unable to detect these low abundant fragments at approximately 19 kDa and instead only detected the main isoform of hemopexin at 75 kDa which showed no trend with Ishak scores (Figure S4). |
| Alpha-1-antichymotrypsin (gene SERPINA3) | Was decreased and has already been observed in our previous study.^6^ |
| Alpha-1-antitrypsin (gene SERPINA1) | Has already been shown to decrease in hepatic scarring.^5^ Our data agrees with this decrease and also shows some isoforms increasing in cirrhosis. |
| Complement C4  and  C4b-binding protein beta chain, | Two proteins involved in the complement cascade which were decreased in cirrhosis. In our previous study we also observed a decrease in complement C4.^6^ C4b-binding protein is composed of alpha chains and a beta chain.^7^ The decrease in specifically the beta chain of C4b-binding protein has not been described in hepatic scarring although total C4b-binding protein has been shown to decrease in cirrhosis.^8^ The beta chain of C4b-binding protein, but not the alpha chain, is expressed in fibroblast-like ovarian cells and may play a role in healing and fibrosis resorption.^7^ If in a similar way the beta chain of C4b-binding protein, but not the alpha chain, is decreasing in hepatic scarring then this may serve as a better biomarker than the already described total C4b-binding protein. Antibodies for C4b-binding protein beta chain were used for Western blotting but were unsuccessful in detecting bands. |

**References**

1. Moriya K, Shintani Y, Fujie H, Miyoshi H, Tsutsumi T, Yotsuyanagi H, Iino S, Kimura S, Koike K. Serum lipid profile of patients with genotype 1b hepatitis C viral infection in Japan. Hepatol Res 2003;25:371-376.

2. Yokoyama Y, Terai S, Ishikawa T, Aoyama K, Urata Y, Marumoto Y, Nishina H, Nakamura K, Okita K, Sakaida I. Proteomic analysis of serum marker proteins in recipient mice with liver cirrhosis after bone marrow cell transplantation. Proteomics 2006;6:2564-70.

3. Benga WJ, Krieger SE, Dimitrova M, Zeisel MB, Parnot M, Lupberger J, Hildt E, Luo G, McLauchlan J, Baumert TF, Schuster C. Apolipoprotein E interacts with hepatitis C virus nonstructural protein 5A and determines assembly of infectious particles. Hepatology 2010;51:43-53.

4. Corpechot C, Benlian P, Barbu V, Chazouilleres O, Poupon RE, Poupon R. Apolipoprotein E polymorphism, a marker of disease severity in primary biliary cirrhosis? J Hepatol 2001;35:324-8.

5. Cheung KJ, Tilleman K, Deforce D, Colle I, Van Vlierberghe H. The HCV serum proteome: a search for fibrosis protein markers. J Viral Hepat 2009;16:418-29.

6. Gangadharan B, Antrobus R, Dwek RA, Zitzmann N. Novel serum biomarker candidates for liver fibrosis in hepatitis C patients. Clin Chem 2007;53:1792-9.

7. Criado-Garcia O, Fernaud-Espinosa I, Bovolenta P, Sainz de la Cuesta R, Rodriguez de Cordoba S. Expression of the beta-chain of the complement regulator C4b-binding protein in human ovary. Eur J Cell Biol 1999;78:657-64.

8. Tamei H, Hoshino T, Yoshida S, Hayashi T, Iwata K, Suzuki K. One-step sandwich enzyme immunoassays for human C4b-binding protein (C4BP) and protein S-C4BP complex using monoclonal antibodies. Clin Chim Acta 1995;234:115-25.
